# Ras-ERK-ETS inhibition alleviates neuronal mitochondrial dysfunction by reprogramming mitochondrial retrograde signaling

**DOI:** 10.1371/journal.pgen.1007567

**Published:** 2018-07-30

**Authors:** Olivia F. Duncan, Lucy Granat, Ramya Ranganathan, Vandana K. Singh, David Mazaud, Manolis Fanto, David Chambers, Clive G. Ballard, Joseph M. Bateman

**Affiliations:** 1 Maurice Wohl Clinical Neuroscience Institute, King’s College London, London, United Kingdom; 2 Wolfson Centre for Age-Related Diseases, King’s College London, London, United Kingdom; 3 Medical School Building, St Luke's Campus, University of Exeter, Exeter, United Kingdom; Stanford University School of Medicine, UNITED STATES

## Abstract

Mitochondrial dysfunction activates the mitochondrial retrograde signaling pathway, resulting in large scale changes in gene expression. Mitochondrial retrograde signaling in neurons is poorly understood and whether retrograde signaling contributes to cellular dysfunction or is protective is unknown. We show that inhibition of Ras-ERK-ETS signaling partially reverses the retrograde transcriptional response to alleviate neuronal mitochondrial dysfunction. We have developed a novel genetic screen to identify genes that modify mitochondrial dysfunction in *Drosophila*. Knock-down of one of the genes identified in this screen, the Ras-ERK-ETS pathway transcription factor Aop, alleviates the damaging effects of mitochondrial dysfunction in the nervous system. Inhibition of Ras-ERK-ETS signaling also restores function in *Drosophila* models of human diseases associated with mitochondrial dysfunction. Importantly, Ras-ERK-ETS pathway inhibition partially reverses the mitochondrial retrograde transcriptional response. Therefore, mitochondrial retrograde signaling likely contributes to neuronal dysfunction through mis-regulation of gene expression.

## Author summary

Loss of mitochondrial function activates the mitochondrial retrograde signaling pathway resulting in large scale changes in nuclear gene transcription. Very little is known about retrograde signaling in the nervous system and how the transcriptional changes affect neuronal function. Here we identify Ras-ERK-ETS signaling as a novel mitochondrial retrograde signaling pathway in the *Drosophila* nervous system. Inhibition of Ras-ERK-ETS signaling improves neuronal function in *Drosophila* models of mitochondrial disease. Targeting Ras-ERK-ETS signaling may therefore have therapeutic potential in mitochondrial disease patients. Using a transcriptomic approach, we find that inhibition of Ras-ERK-ETS signaling partially reverses the mitochondrial retrograde transcriptional response. Surprisingly therefore, the mitochondrial retrograde transcriptional response contributes to neuronal dysfunction.

## Introduction

The use of ATP as a universal currency of energy transfer makes this molecule essential for life. ATP is generated either by glycolysis in the cytosol, or through the action of the tricarboxylic acid (TCA) cycle and β-oxidation of fatty acids coupled to oxidative phosphorylation (OXPHOS) in mitochondria. The mitochondrial electron transport chain (ETC) couples the transfer of electrons to the pumping of protons into the inter-membrane space [1]. This creates a membrane potential (ΔΨ), which is used by the mitochondrial ATP synthase to convert ADP to ATP [2]. Under normal aerobic conditions mitochondria use OXPHOS to generate the majority of cellular ATP. Mitochondria also metabolize fatty acids, synthesize amino acids, buffer cellular calcium ions (Ca^2+^), produce the majority of cellular reactive oxygen species, synthesise iron-sulphur clusters and mediate programmed cell death.

Mitochondria are abundant in almost every cell type and are particularly important in the nervous system. During nervous system development neural stem cell progenitors use mainly glycolytic metabolism, but upon differentiation into neurons switch to become dependent on OXPHOS for ATP [3, 4]. Mature neurons must maintain their membrane potential through the action of ATP dependent sodium potassium pumps and so are vitally dependent on mitochondrial function. Failure of mitochondria and breakdown of the cellular power supply can lead to human disease [5, 6]. Primary mitochondrial diseases are caused by mutations in mitochondrial genes, either in the nuclear or mitochondrial genome [7]. These diseases are rare, but can be severe and debilitating, often affecting the nervous system or muscle. The mitochondrial disease Leigh syndrome, caused by mutations in subunits of mitochondrial ETC complex I or IV, causes degeneration of the nervous system and death in infants [8].

Mitochondrial dysfunction is also strongly implicated in neurodegenerative diseases and may be a common pathway in Alzheimer’s disease, Parkinson’s disease, Amyotrophic lateral sclerosis and Huntington’s disease [9]. Reduced mitochondrial ETC complex I activity in the substantia nigra pars compacta is a hallmark of Parkinson’s disease and toxins that inhibit complex I cause dopaminergic neuron cell death and parkinsonian phenotypes in humans and rodent models [10–14]. Moreover, mutations in two genes involved in mitochondrial quality control, *PINK1* and *Parkin*, cause familial Parkinson’s disease [15]. There is also evidence supporting a role for mitochondrial dysfunction in Alzheimer’s, as patients have been shown to have mitochondrial ETC complex IV and V deficits and mitochondrial DNA mutations have been associated with this disease [16–20].

Cells respond to changes in mitochondrial function by altering nuclear gene expression, a process known as mitochondrial retrograde signaling [21–23]. The mechanism of retrograde signaling varies depending on the organism and cell type. In yeast the retrograde response is triggered by decreased glutamate, whose synthesis is reduced due to failure of the TCA cycle to synthesise α-ketoglutarate, the precursor of glutamate [24]. This retrograde signaling pathway culminates in the partial dephosphorylation of the transcription factor Rtg3 which, together with its binding partner Rtg1, relocalises to the nucleus to activate gene expression [25]. In other contexts, mitochondrial dysfunction can trigger a retrograde signaling pathway known as the mitochondrial unfolded protein response (UPR^mt^). When activated, the UPR^mt^ induces the expression of nuclear encoded mitochondrial chaperone proteins and proteases [26]. In *C*. *elegans*, activating transcription factor associated with stress-1 (ATFS-1) is normally localised to mitochondria, but upon mitochondrial dysfunction a fraction of ATFS-1 localises to the nucleus where it regulates the expression of UPR^mt^ genes [27, 28].

*ATFS-1* is not conserved in mammals and the *RTG* genes are not present in metazoans, but analogous retrograde signaling pathways have been identified that enable mitochondria to reprogram nuclear gene expression [29, 30]. Transcriptional studies have shown that mitochondrial dysfunction elicits large scale changes in nuclear gene expression in diverse cell types in a range of model systems [22, 23, 31]. However, the molecular basis of mitochondrial signaling is still poorly understood, particularly in the nervous system [29, 30]. Importantly, it is not known whether the retrograde transcriptional response contributes to mitochondrial dysfunction phenotypes or is protective. To address these questions, we performed a genetic screen and identified 30 genes that modify mitochondrial dysfunction in the *Drosophila* wing, implicating several new pathways in the mitochondrial retrograde response. Manipulation of one of the identified pathways, Ras-ERK-ETS signaling, also alleviates the effects of mitochondrial dysfunction in the *Drosophila* nervous system. Transcriptomic and functional analyses suggest that mitochondrial retrograde signaling is reversed and transcriptionally reprogrammed by Ras-ERK-ETS inhibition to restore neuronal function.

## Results

### Knock-down and overexpression of TFAM cause mitochondrial dysfunction and activate mitochondrial retrograde signaling in the *Drosophila* wing

**Fig 1:**
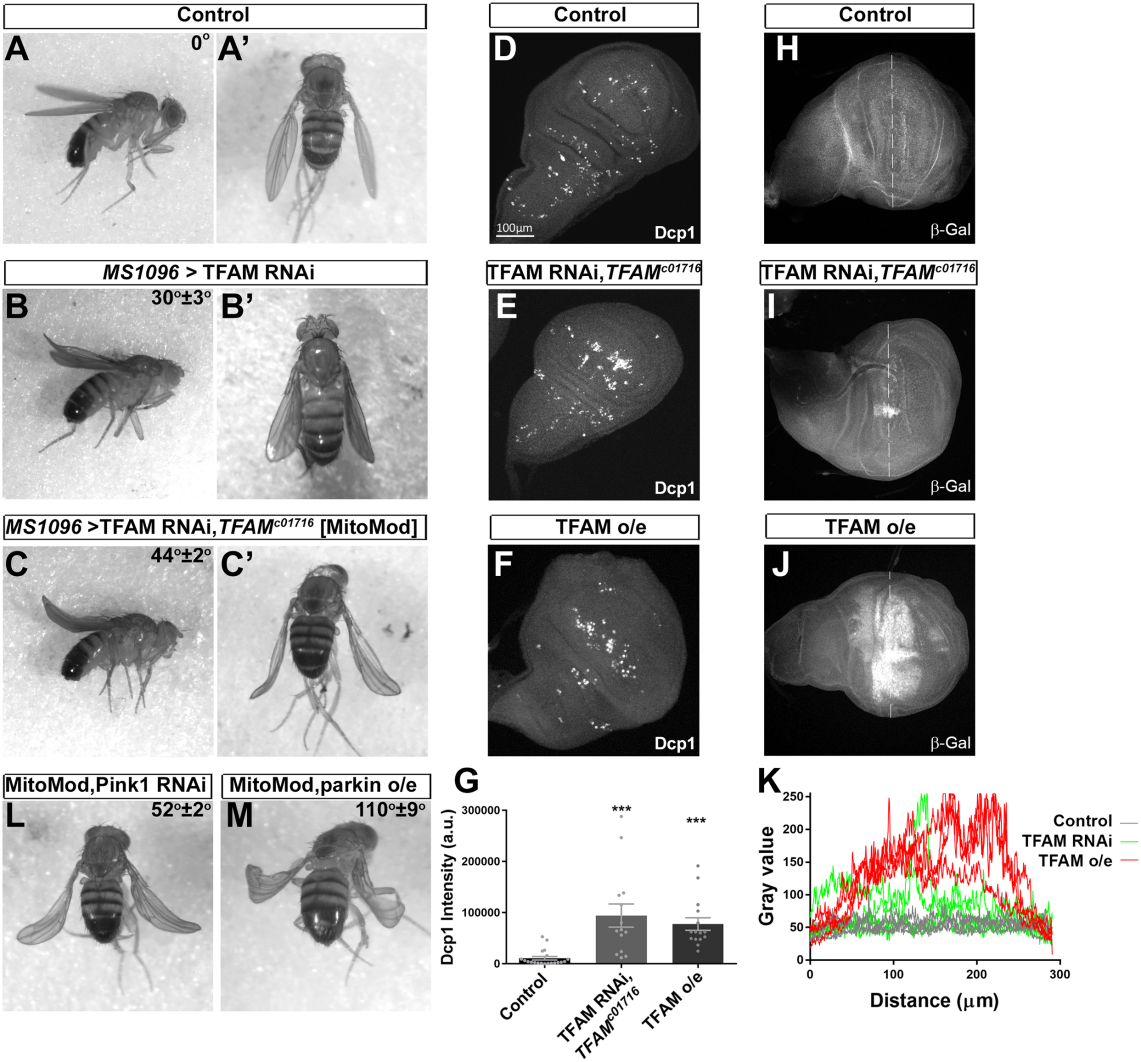
Knock-down of TFAM in the wing causes a mitochondrial dysfunction sensitised phenotype. (A) A control male fly hemizygous for *MS1096-Gal4* has straight wings. (B) Knock-down of TFAM (4217R-1) in the dorsal compartment of the wing using *MS1096-Gal4* causes a slightly curved wing in male flies. (C) Knock-down of TFAM (4217R-1) using *MS1096-Gal4* in a male fly heterozygous for *TFAM*^*c01716*^ enhances the wing curve to approximately 45°. (D-F) Compared to a wing disc from a control larva hemizygous for *MS1096-Gal4* (D), cleaved *Drosophila* effector caspase (Dcp1) expression is increased by knock-down of TFAM (4217R-1) in a *TFAM*^*c01716*^ heterozygous background (E) and by TFAM overexpression (F) in the dorsal compartment of the wing disc where *MS1096-Gal4* expression is highest. (G) Quantification of Dcp1 expression. (H-J) β-Gal antibody staining for *Thor-lacZ* expression in a wing disc from a control larva hemizygous for *MS1096-Gal4* (H), or with knock-down of TFAM (4217R-1) in a *TFAM*^*c01716*^ heterozygous background (I), or TFAM overexpression (J). (K) β-Gal staining intensity (gray value) along a line placed across the dorsal compartment (dashed lines in H-J) for four wing discs for each condition. Data are represented as mean +/- SEM, ***p≤0.001. (L,M) Knock-down of Pink1 (JF01672) (L), or overexpression of parkin (M), enhances the curved wing phenotype in MitoMod flies. Average wing tip angles (n = 3–4) ± S.D are shown, Pink1 knock-down and parkin overexpression phenotypes are significantly different to the MitoMod control (p<0.01).

Mitochondrial retrograde signaling is activated in response to mitochondrial dysfunction. To induce mitochondrial dysfunction, we manipulated the expression levels of the mitochondrial DNA binding protein/transcription factor TFAM. TFAM expression is essential for mitochondrial DNA maintenance (S1A Fig) and gene expression, but overexpression of TFAM in mice and human cells also causes reduced mitochondrial gene transcription and mitochondrial dysfunction [32–35]. In *Drosophila*, both ubiquitous TFAM knock-down (S1B–S1E Fig) or TFAM overexpression [36] cause reduced mitochondrial gene expression and lethality at the larval stage. To develop a rapid, genetically modifiable assay for mitochondrial dysfunction in vivo we tested TFAM knock-down or TFAM overexpression in the wing using *MS1096-Gal4*. Strong TFAM overexpression in the wing causes late pupal lethality, while weak TFAM overexpression or TFAM knock-down (using several independent RNAi transgenes) results in a curved adult wing phenotype (Fig 1B, S1F, S1G, S1J–S1L Fig). Importantly, the TFAM knock-down curved wing phenotype is enhanced by heterozygosity for a loss-of-function mutation in *TFAM* (*TFAM*^*c01716*^) (Fig 1C, S1H Fig) and almost completely rescued by co-expression of TFAM (S1M–S1P Fig), proving that this RNAi phenotype is a result of reduced TFAM expression. Although neither TFAM knock-down or overexpression change ATP levels (S2A–S2K Fig), or reactive oxygen species (ROS) (S2L–S2S Fig) in the developing wing, both cause reduced mitochondrial numbers and increased apoptosis (Fig 1D–1G, S2T–S2X Fig).

We previously showed that the gene *Thor*, encoding the eukaryotic initiation factor 4E binding protein (4E-BP), is a mitochondrial retrograde signaling response gene in neurons [36]. Both knock-down and overexpression of TFAM in the wing imaginal disc cause increased *Thor* expression (Fig 1H–1K). TFAM overexpression causes a strong increase in *Thor* expression throughout and beyond the dorsal compartment, while TFAM knock-down causes increased *Thor* expression in discreet patches within the dorsal compartment (Fig 1H–1K). As a result, using qRT-PCR from whole wing discs we could only detect increased *Thor* expression, or increased expression of the mitochondrial unfolded response pathway target gene *Hsp22* [37] with TFAM overexpression (S2Y and S2Z Fig). These data show that mitochondrial dysfunction caused by TFAM knock-down and TFAM overexpression activate mitochondrial retrograde signaling in the wing.

In order to perform a genetic screen for modifiers of mitochondrial retrograde signaling, flies were generated that stably express the TFAM RNAi transgene, together with the *TFAM*^*c01716*^ mutation, in the wing. These flies, referred to as ‘MitoMod’ for ‘Mitochondrial Modifier’, have a distinctive ~45° curve at the tip of the wing (Fig 1C). To test the sensitivity of MitoMod flies to mitochondrial perturbation they were crossed to lines carrying RNAi or overexpression transgenes for genes associated with familial Parkinson’s disease that have mitochondrial associated functions. Transgenes that cause a wing phenotype when expressed on their own were excluded to avoid additive effects (S1 Table). Overexpression or knock-down of *Pink1*, overexpression of *parkin*, knock-down of *Lrrk* and overexpression and knock-down of *DJ-1α* and *DJ-1β* all enhance the MitoMod wing phenotype (Fig 1L and 1M, S3 Fig and S1 Table). Therefore, the MitoMod wing phenotype provides a sensitised background for identifying mitochondrial retrograde signaling genes *in vivo*.

### A genetic screen for genes that modify mitochondrial retrograde signaling identifies Aop

**Fig 2:**
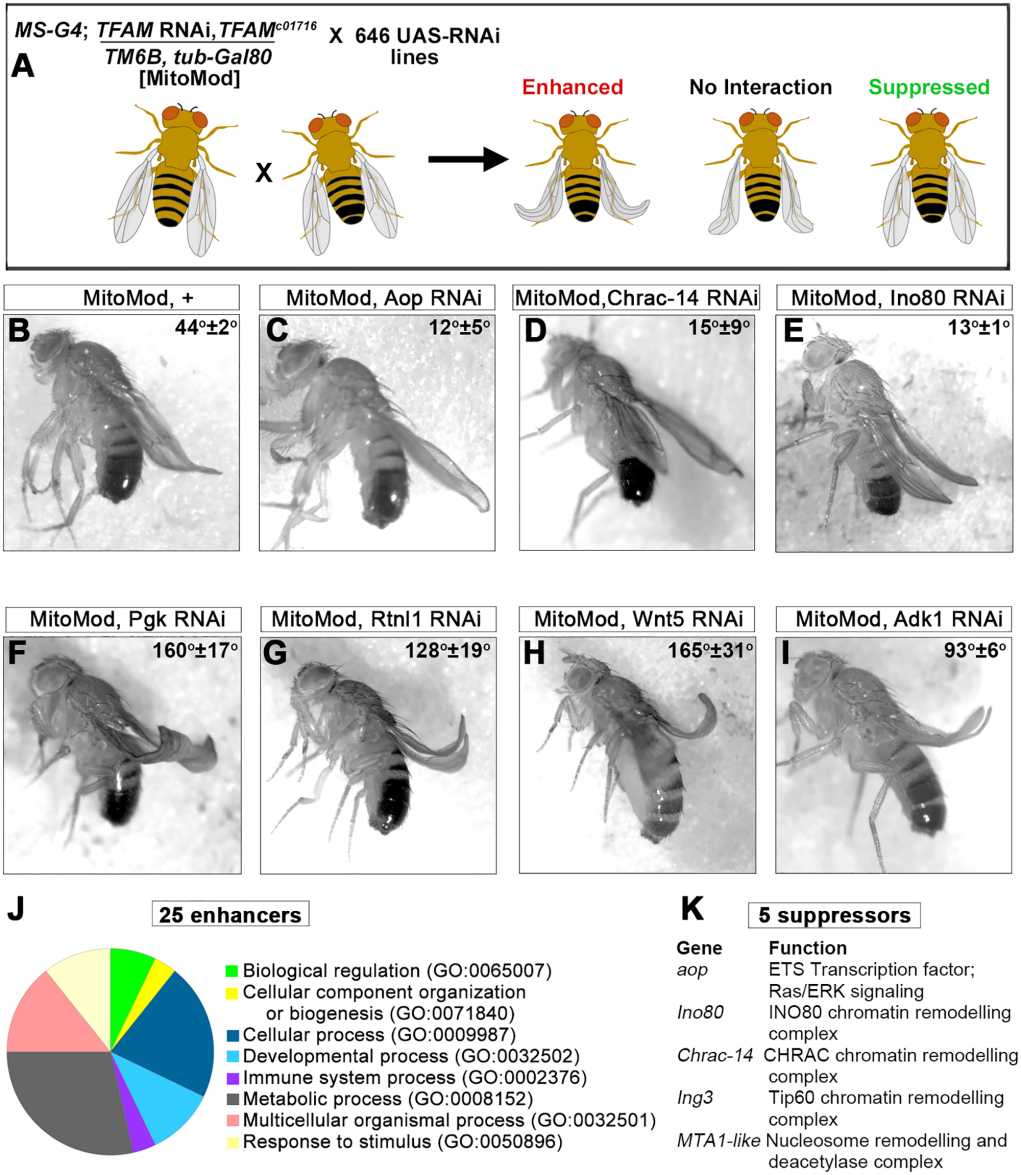
A genetic modifier screen for the cellular response to mitochondrial dysfunction. (A) Schematic of the genetic screen methodology, see text for details. (B) Knock-down of TFAM (4217R-1) in a mutant *TFAM*^*c01716*^ heterozygous background in MitoMod flies causes a 45° curve at the wing tip. (C-E) Knock-down of Anterior open (Aop, 3166R-1) (C), chromatin assembly complex 15kDa protein (Chrac-14; 13399R-6) (D) and INO80 complex subunit (Ino80; GL00616) (E) suppress the MitoMod phenotype. (F-I) Knock-down of phosphoglycerate kinase (Pgk; 110081) (F), Reticulon-like 1 (Rtnl1; 110545) (G), Wnt oncogene analog 5 (Wnt5; 101621) (H) and Adenylate kinase 1 (Adk1; GL00177) (I) enhance the MitoMod phenotype. Average wing tip angles (n = 3–4) ± S.D are shown, all modifier gene wing angles are significantly different to the MitoMod control (p<0.01). (J) Pie chart showing the GO biological process of the genes which enhance the MitoMod phenotype. (K) Genes which suppress the MitoMod phenotype.

MitoMod flies were used to perform a genetic modifier screen of 646 RNAi lines, targeting 579 genes (Fig 2A). This RNAi collection was enriched for lines that target genes expressed in the nervous system and genes encoding chromatin remodelling factors (S2 Table). RNAi lines that cause a phenotype when expressed alone in the wing were excluded (S2 Table), to avoid additive effects with the MitoMod wing phenotype. Stringent criteria were used to identify interacting genes: only RNAi lines that caused a strong reproducible enhancement or suppression, which was also replicated by an independent RNAi line targeting the same gene were classed as hits. 25 genes were identified that enhance the MitoMod wing phenotype (Fig 2F–2I, S3 Table). Gene ontology (GO) analysis shows that these genes are involved in a range of biological processes and functions (Fig 2J, S3 Table). Five genes were identified that suppress the MitoMod phenotype (Fig 2C–2E, S4 Table). The suppressor genes function in chromatin remodelling or transcriptional regulation (Fig 2K, S4 Table). Overall, the variety in function of genes identified in the screen is consistent with the multifunctional cellular roles of mitochondria.

To test whether suppression of the mitochondrial dysfunction phenotype in the adult wing reflects reduced apoptosis, cleaved *Drosophila* effector caspase (Dcp-1) expression was analysed in the wing during development. Knock-down of TFAM in the wing causes increased Dcp-1 expression, but this is reduced to control levels by knock-down of the suppressors identified in the screen Aop, Ino80, Chrac-14, Ing3 and MTA1-like (S4A–S4I Fig). Moreover, knock-down of Aop or Ino80 suppress the increase in apoptosis caused by TFAM overexpression (S4J Fig). These data show that suppression of the adult wing phenotype in MitoMod flies reflects a reduction in apoptosis.

### Inhibition of Ras-ERK-ETS signaling improves function in neurons with a mitochondrial deficit

The effects of mitochondrial dysfunction are particularly acute in the nervous system and manipulation of retrograde signaling may be a potential strategy to alleviate these effects. We aimed to use the wing screen to identify genes involved in mitochondrial retrograde signaling in the nervous system. Aop (anterior open, also known as Yan), one of the suppressor genes identified (Fig 2K), is an E-twenty six (ETS) transcription factor and a target of the highly conserved Ras-ERK (mitogen-activated protein kinase (MAPK)) pathway. Treatment of cultured neuronal cells with the mitochondrial uncoupler carbonyl cyanide p-(trifluoromethoxy) phenylhydrazone (FCCP) causes aberrant ERK activation [38]. We therefore hypothesised that Ras-ERK signaling is mis-regulated by mitochondrial dysfunction in the nervous system and that manipulation of Ras-ERK signaling would modify the effects of neuronal mitochondrial dysfunction.

**Fig 3:**
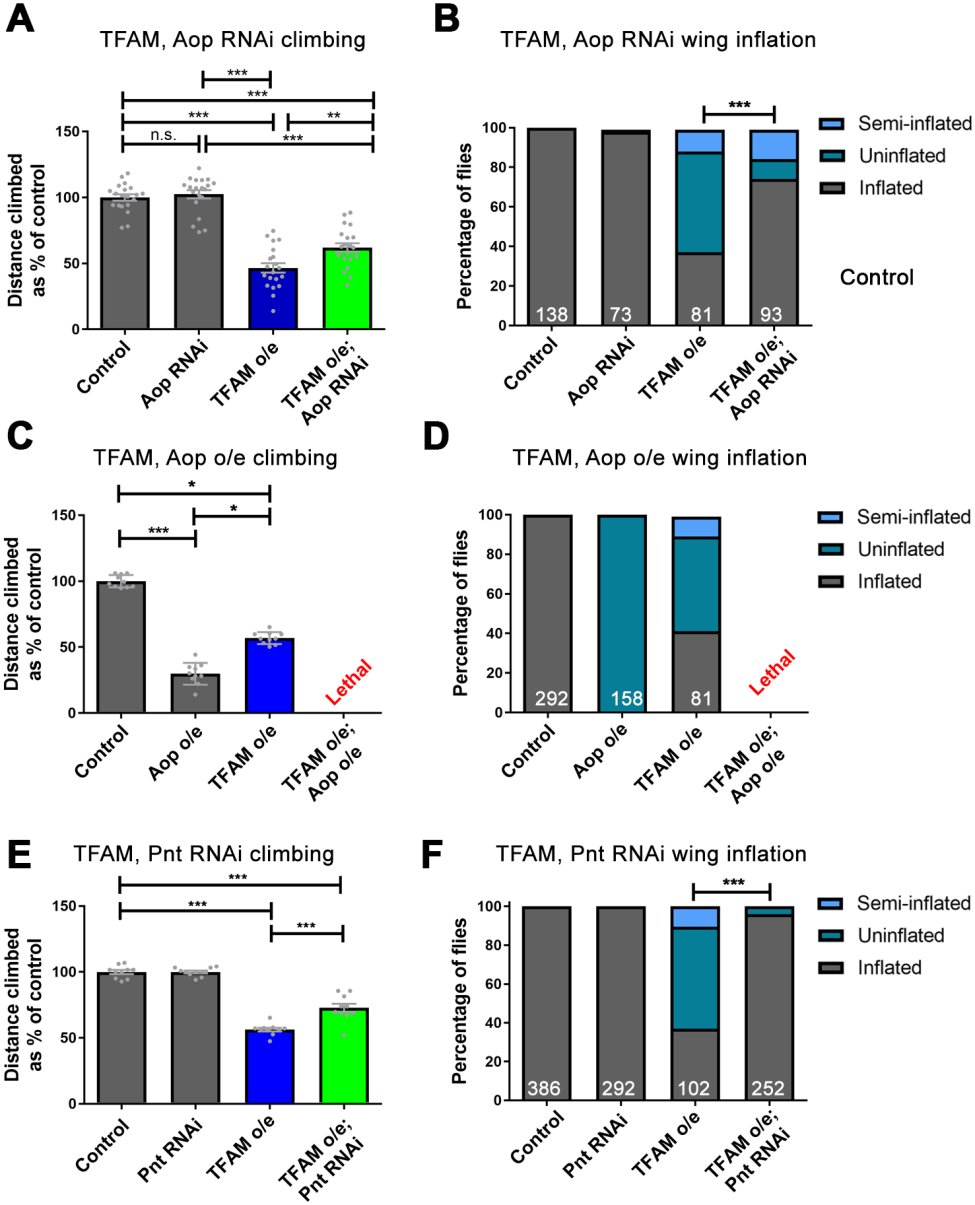
Knock-down of Aop and Pnt suppress the mitochondrial dysfunction phenotype in neurons. (A,B) Knock-down of Aop (3166R-1) suppresses the climbing (A) and wing inflation phenotype (B) caused by overexpression of TFAM using *D42-Gal4*. (C,D) Aop overexpression with *D42-Gal4* causes a severe climbing (C) and wing inflation phenotype (D) and lethality when overexpressed in combination with TFAM. (E,F) Knock-down of Pnt (JF02227) suppresses the climbing (E) and wing inflation phenotype (F) caused by overexpression of TFAM with *D42-Gal4*. Data are represented as mean +/- SEM, *p≤0.05, **p≤0.01, ***p≤0.001. For wing inflation assays the numbers of flies counted for each genotype are shown in white. Controls are *D42-Gal4* hemizygotes.

TFAM knock-down in the wing causes similar but weaker effects to TFAM overexpression (S1J–S1L Fig). We have previously shown that TFAM overexpression in motor neurons causes a dramatic reduction in pre-synaptic tetramethylrhodamine methyl ester (TMRM) positive and mitochondrial-GFP labelled mitochondrial number and volume and a robust adult climbing phenotype [36]. Overexpression of TFAM in neurons with *D42-Gal4* also causes a failure of wing inflation in approximately 50% of flies, due to dysfunction of the CCAP neurons that release the neuropeptide bursicon, which activates wing inflation (S5L–S5N Fig) [36]. TFAM knock-down in motor neurons also causes a reduction in mitochondrial volume and reduced adult climbing, but these phenotypes are much weaker than with TFAM overexpression (S5A–S5G Fig) and TFAM knock-down does not affect wing inflation. We therefore used TFAM overexpression as a model to test whether targeting Ras/ERK signaling modifies the effects of mitochondrial dysfunction in neurons. Consistent with the suppressive effect in the screen, knock-down of Aop in neurons (using a validated RNAi transgene (S5H–S5K Fig)) suppresses the TFAM overexpression climbing and wing inflation phenotypes (Fig 3A and 3B). Furthermore, knock down of the Ras-ERK pathway components Downstream of raf1 (Dsor; MAPK) or the MAPK Rolled (Rl; MAPK kinase) both suppressed the TFAM overexpression climbing and wing inflation phenotypes (S5O–S5V Fig), confirming that inhibition of Ras-ERK signalling alleviates the effects of neuronal mitochondrial dysfunction.

To test whether increasing Aop affects neuronal function we overexpressed Aop alone in motor neurons, or together with TFAM. Overexpression of Aop in neurons causes a strong climbing deficit and complete failure of wing inflation (Fig 3C and 3D). Moreover, overexpression of Aop and TFAM together causes pupal lethality (Fig 3C and 3D), which is not affected by heterozygosity for the *Ras85D* loss-of-function allele *Ras85D*^*ΔC40B*^, consistent with Aop acting downstream of Ras.

The ETS domain transcription factor pointed (Pnt) is a second target of Ras-ERK signaling in *Drosophila*. To test whether reducing Pnt expression also modifies neuronal mitochondrial dysfunction we used both a validated RNAi targeting Pnt (S6A–S6D Fig) and heterozygosity for a loss-of-function mutation in *pnt* (*pnt*^*Δ88*^). Knock-down of Pnt in motor neurons, or heterozygosity for *pnt*, suppresses the TFAM overexpression climbing and wing inflation phenotypes (Fig 3E and 3F, S6E and S6F Fig). Combining Pnt knock-down with heterozygosity for *Ras85D* causes significantly increased suppression of TFAM overexpression phenotypes compared to either condition alone (S6G and S6H Fig). However, this is not the case for Aop knock-down and *Ras85D*^*ΔC40B*^ (S6I and S6J Fig). Simultaneous knock-down of Pnt and Aop with TFAM overexpression does not significantly improve the climbing phenotype, but significantly improves the wing inflation phenotype compared to TFAM overexpression with Aop knock-down (S6K and S6L Fig). PntP2 overexpression in motor neurons causes a climbing deficit and lethality in combination with TFAM overexpression (S6M Fig), which is not affected by heterozygosity for the *Ras85D* loss-of-function allele *Ras85D*^*ΔC40B*^, consistent with Pnt acting downstream of Ras. Therefore, reduced expression of the Ras-ERK pathway components Aop and Pnt partially overcomes the damaging effects of mitochondrial dysfunction in motor neurons.

Aop and Pnt are the main transcriptional effectors of the Ras-ERK pathway. To test whether directly activating Ras modulates neuronal activity in neurons with inhibited mitochondrial function we used a constitutively active form of Ras, Ras^V12^. Expression of *Ras85D*^*V12*^ in motor neurons causes lethality with *D42-Gal4* and with *OK371-Gal4*. Knock-down of either Aop or Pnt suppresses the Ras85D^V12^ lethality phenotype (S6N and S6O Fig), while Ras85D^V12^ combined with Aop overexpression is lethal. Therefore, Ras-ERK pathway activation mimics the effects of mitochondrial dysfunction and Aop and Pnt both act as positive regulators of the pathway in motor neurons.

**Fig 4:**
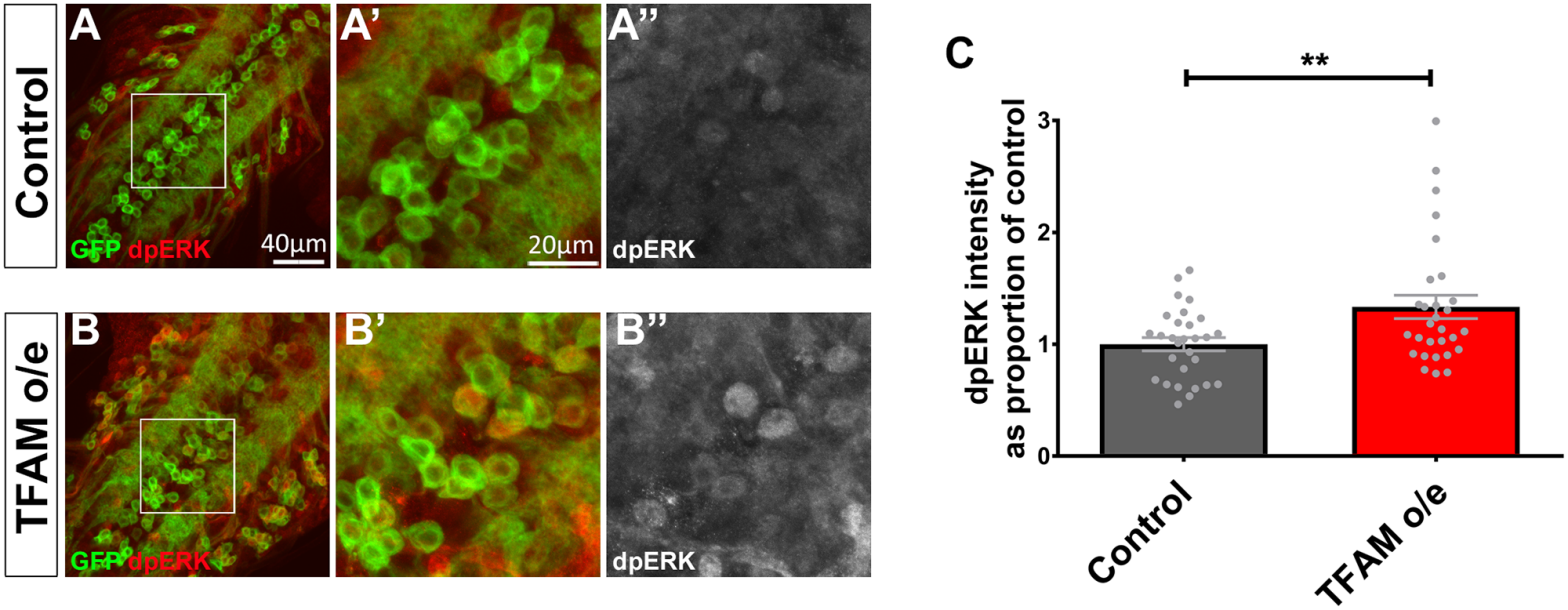
Neuronal mitochondrial dysfunction causes increased dpERK expression. (A) The VNC from a control larva expressing CD8-GFP (green) in motor neurons using *OK371-Gal4* stained for dpERK expression (red in A,A’,B,B’, white in A”, B”). (B) The VNC from a larva overexpressing TFAM using *OK371-Gal4* shows increased dpERK expression in motor neurons. (C) Quantification of dpERK expression in motor neuron cell bodies. Data are represented as mean +/- SEM, **p≤0.01.

We next looked for direct evidence of Ras-ERK-ETS pathway mis-regulation in neurons caused by mitochondrial dysfunction. Motor neurons overexpressing TFAM were stained with an antibody that recognises the activated (di-phosphorylated) form of ERK (dpERK). dpERK expression is significantly increased in larval motor neurons overexpressing TFAM (Fig 4). Clonal analysis of TFAM overexpression shows that activation of dpERK is both cell autonomous and non-cell autonomous (S6P and S6Q Fig). Therefore, Ras-ERK-ETS signaling is activated in response to mitochondrial dysfunction in *Drosophila* neurons.

### Knock-down of Pnt rescues the active zone phenotype caused by mitochondrial dysfunction

**Fig 5:**
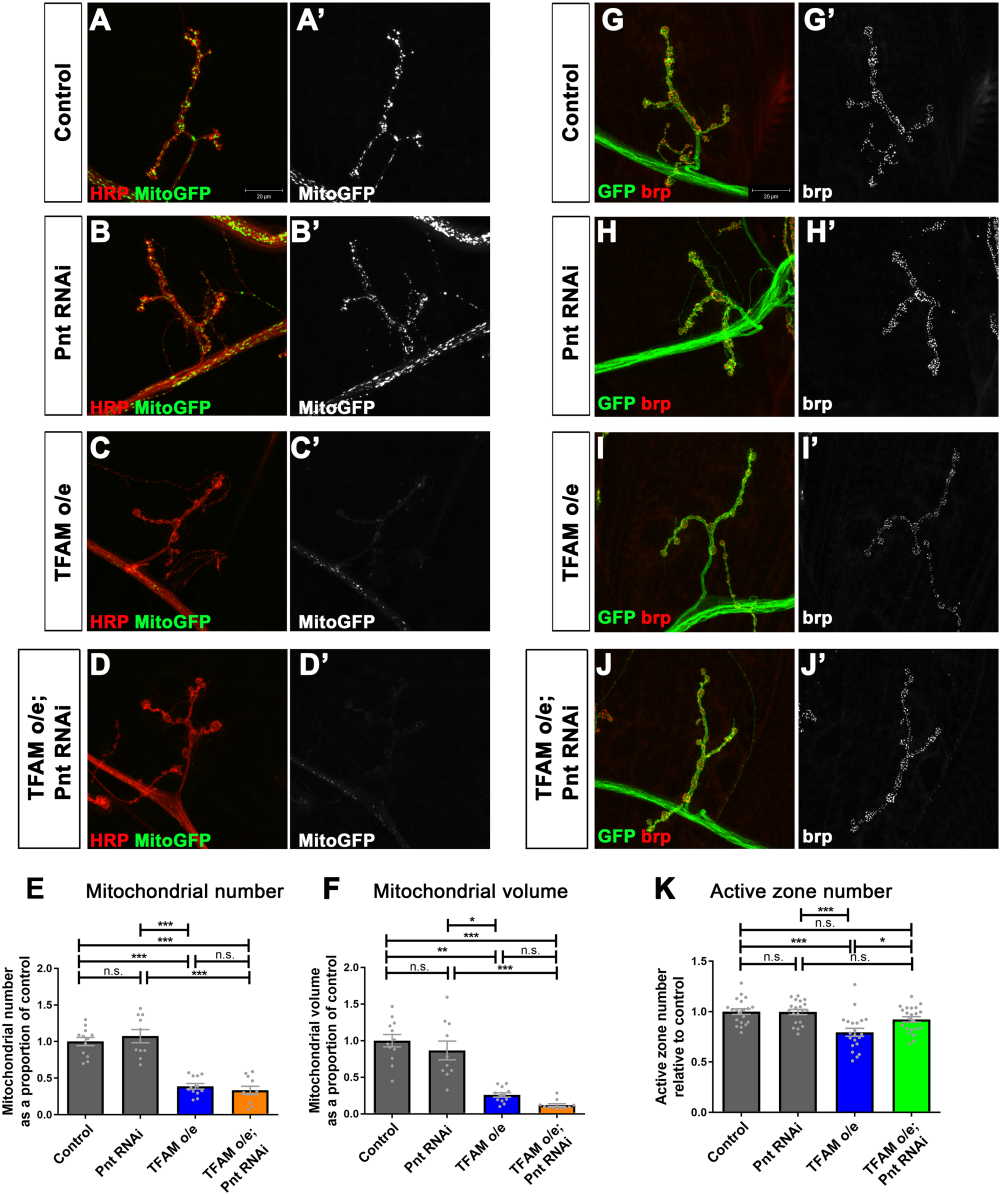
Knock-down of Pnt rescues the reduction in active zones caused by mitochondrial dysfunction. (A-D) Segment A3, muscle 4 NMJ in late third instar larvae from control (A), or with Pnt RNAi (JF02227) (B), TFAM overexpression (C), Pnt RNAi (JF02227) and TFAM overexpression (D) in motor neurons using *OK371-Gal4*. Motor neuron specific expression of mitoGFP (green in (A-D) and white in (A’-D’)) was used to visualise mitochondria and staining for horse radish peroxidase (red) to visualise neuronal membranes. (E,F) Quantification of mitochondrial number (E) and volume (F). (G-J) Segment A3, muscle 4 NMJ in late third instar larvae from control (G), Pnt RNAi (JF02227) (H), TFAM overexpression (I), or Pnt RNAi (JF02227) and TFAM overexpression (J) in motor neurons using *OK371-Gal4*. Expression of CD8-GFP (GFP, green) is used to visualise neuronal membranes and brp staining (red in G-J and white in G’-J’) to visualise active zones. (K) Quantification of active zone number. Data are represented as mean +/- SEM, n.s. not significant, *p≤0.05, **p≤0.01, ***p≤0.001. Controls are *OK371-Gal4* hemizygotes.

Similar to mitochondrial dysfunction in late larval sensory neurons, which does not affect ATP levels due to a compensatory increase in glycolysis [39], overexpression of TFAM in motor neurons does not alter ATP levels (S7A–S7C Fig). Active zones are the sites of pre-synaptic neurotransmitter release at chemical synapses and are enriched for the protein complexes that regulate synaptic vesicle release and recycling. TFAM overexpression in motor neurons causes altered mitochondrial morphology in the cell body, a dramatic loss of pre-synaptic mitochondria and a reduction in the number of active zones at the larval neuromuscular junction (NMJ) [36]. Therefore, to further investigate how modulation of Ras-ERK-ETS signaling affects neuronal mitochondrial dysfunction we focused on the synaptic compartment. Neither knock-down of Aop or Pnt affects the severe loss of pre-synaptic mitochondria caused by TFAM overexpression (Fig 5A–5F. S7D and S7E Fig), suggesting that Ras-ERK-ETS pathway inhibition does not alter the primary mitochondrial defect. Knock-down of Aop does not rescue the active zone phenotype caused by TFAM overexpression, but Aop knock-down alone causes a reduction in active zone number (S7F Fig), complicating the interpretation of this result. However, knock-down of Pnt fully rescues the active zone phenotype caused by TFAM overexpression (Fig 5G and 5K). These data show that Ras-ERK-ETS pathway inhibition does not affect the primary mitochondrial defect, but modifies the active zone phenotype caused by neuronal mitochondrial dysfunction.

### Inhibition of Ras-ERK-ETS signaling improves function in *Drosophila* models of mitochondrial disease and Parkinson’s

**Fig 6:**
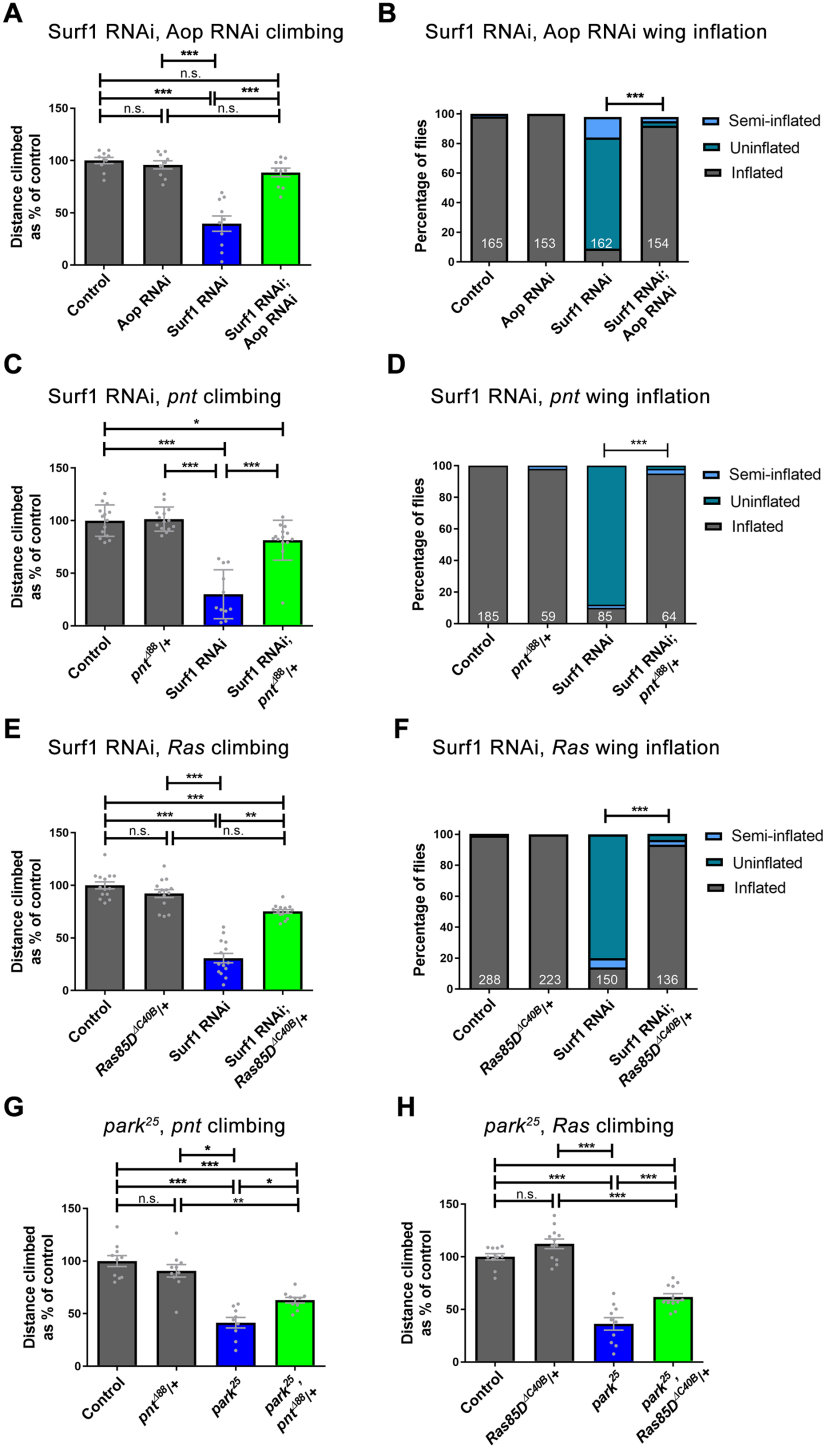
Inhibition of Ras-ERK-ETS signaling improves function in *Drosophila* models of mitochondrial disease and Parkinson’s. (A,B) Knock-down of Aop (3166R-1) suppresses the climbing (A) and wing inflation phenotype (B) caused by pan-neuronal knock-down of Surf1 with *nSyb-Gal4*. (C,D) Heterozygosity for *pnt* (*pnt*^*Δ88*^) suppresses the climbing (C) and wing inflation phenotype (D) caused by knock-down of Surf1 with *nSyb-Gal4*. (E,F) Heterozygosity for *Ras85D* (*Ras85D*^*ΔC40B*^) suppresses the climbing (E) and wing inflation phenotype (F) caused by knock-down of Surf1 with *nSyb-Gal4*. Controls are *nSyb-Gal4* hemizygotes. (G,H) Heterozygosity for *pnt* (G), or *Ras85D* (H) suppresses the climbing phenotype in *park*^*25*^ homozygous flies. Controls are *w*^*1118*^. Data are represented as mean +/- SEM, *p≤0.05, **p≤0.01, ***p≤0.001. For wing inflation assays the numbers of flies counted for each genotype are shown in white.

Mitochondrial dysfunction in humans causes rare primary mitochondrial diseases and is also associated with more common neurodegenerative diseases, including Parkinson’s. We hypothesised that targeting the retrograde response, through inhibition of Ras-ERK-ETS signaling, would be beneficial in *Drosophila* models of human disease associated with mitochondrial dysfunction. To test this we used pan-neuronal (*nSyb-Gal4*) knock-down of the OXPHOS complex IV subunit Surf1, a model for the primary mitochondrial childhood encephalomyelopathy Leigh syndrome, and *park*^*25*^ homozygous mutant flies, a model for familial Parkinson’s disease [36, 40, 41]. dpERK expression is increased in the ventral nerve cord (VNC) by pan-neuronal knock-down of Surf1, but not in *park*^*25*^ homozygous larvae (S7G and S7H Fig), possibly because of the mild effect of loss of Parkin in neurons. Knock-down of Aop, knock-down or heterozygosity for *pnt* all suppress the severe climbing phenotype and rescue the wing inflation defect caused by pan-neuronal knock-down of Surf1 (Fig 6A–6D, S8A and S8B Fig). Knock-down of Aop does not significantly improve climbing in the *park*^*25*^ mutant (S8C Fig). However, heterozygosity for *pnt* does suppress the climbing phenotype in *park*^*25*^ mutant flies (Fig 6G). To test whether the beneficial effects in these two models are specific to reduced Aop and Pnt expression, or a general property of Ras-ERK-ETS pathway inhibition, we used a loss-of-function allele of *Ras85D* (*Ras85D*^*ΔC40B*^). Heterozygosity for *Ras85D* suppresses the climbing and wing inflation defects caused by knock-down of Surf1 (Fig 6E and 6F) and the climbing deficit in *park*^*25*^ homozygous flies (Fig 6H). Thus, targeting the retrograde response, through inhibition of Ras-ERK-ETS signaling, improves function in two independent models of human disease caused by mitochondrial dysfunction.

### Inhibition of Ras-ERK-ETS signaling partially reverses the transcriptional response to neuronal mitochondrial dysfunction

**Fig 7:**
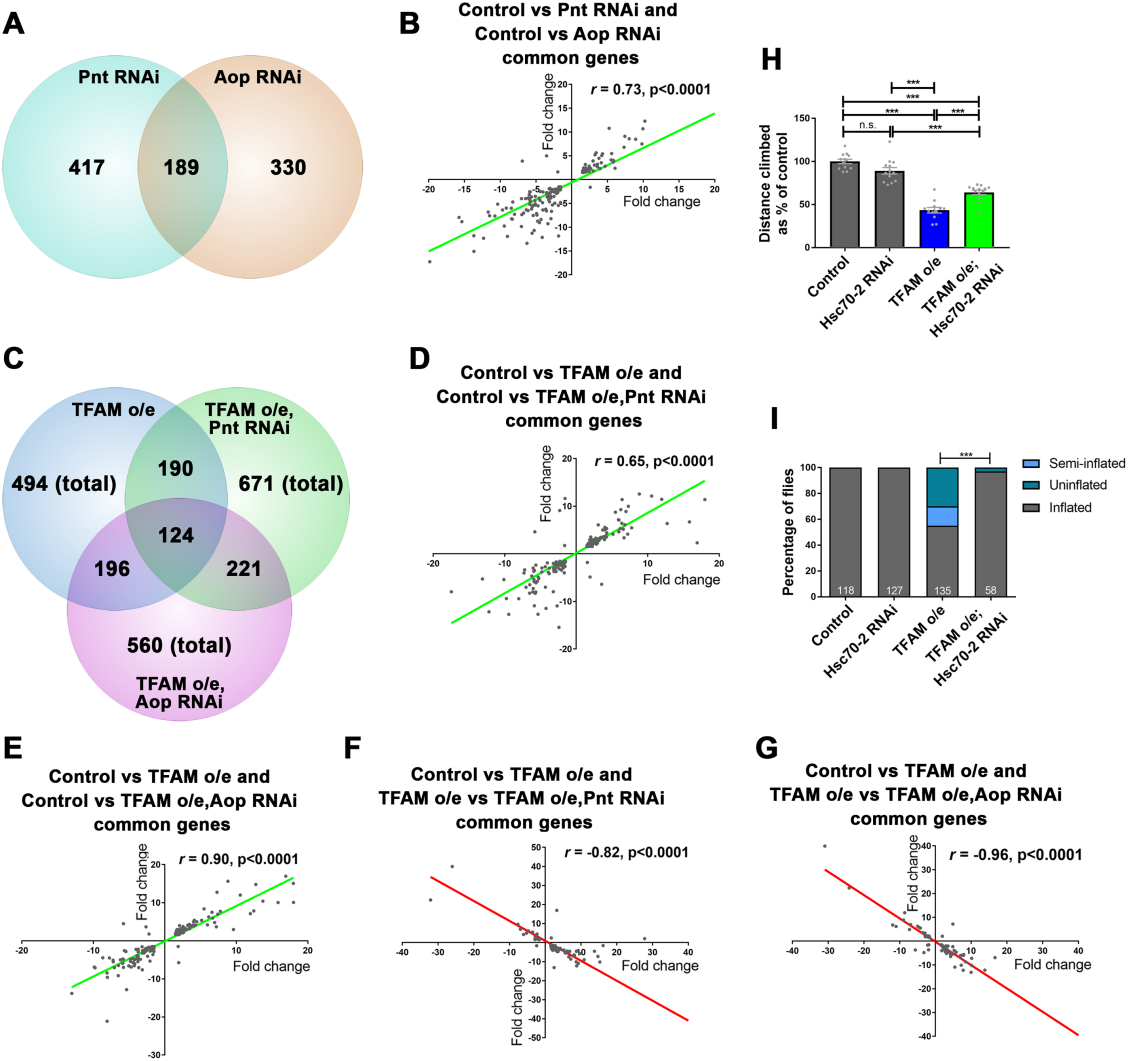
Inhibition of Ras-ERK-ETS signaling partly reverses the transcriptional mis-regulation caused by TFAM overexpression in the nervous system. (A) Numbers of genes that are mis-regulated by Pnt and Aop knock-down versus control. (B) Correlation of expression levels between genes mis-regulated in both Pnt and Aop knock-down conditions. (C) Numbers of genes mis-regulated by TFAM overexpression versus control, TFAM overexpression combined with Pnt knock-down versus control, or TFAM overexpression combined with Aop knock-down versus control. (D) Correlation of expression levels between genes that were mis-regulated in both TFAM overexpression versus control and TFAM overexpression combined with Pnt knock-down versus control conditions. (E) Correlation of expression levels between genes that were mis-regulated in both TFAM overexpression versus control and TFAM overexpression combined with Aop knock-down versus control conditions. (F) Correlation of expression levels between genes that were mis-regulated in both TFAM overexpression versus control and TFAM overexpression versus TFAM overexpression combined with Pnt knock-down conditions. (G) Correlation of expression levels between genes that were mis-regulated in both TFAM overexpression versus control and TFAM overexpression versus TFAM overexpression combined with Aop knock-down conditions. (H,I) Knock-down of Hsc70-2 suppresses the climbing (H) and wing inflation (I) phenotypes caused by TFAM overexpression in motor neurons using *D42-Gal4*. Data are represented as mean +/- SEM, n.s. not significant, ***p≤0.001. For wing inflation assays the numbers of flies counted for each genotype are shown in white.

Mitochondrial dysfunction transcriptionally reprograms cells by altering nuclear gene expression [22, 29]. It is not known whether this transcriptional reprogramming is protective or damaging. Ras-ERK-ETS pathway inhibition could benefit neurons either by enhancing the retrograde transcriptional response, or by reversing the expression of genes that are mis-regulated in response to mitochondrial dysfunction. To understand the mechanism by which Ras-ERK-ETS signaling alleviates the effects of neuronal mitochondrial dysfunction, we performed transcriptomic analysis using central nervous system (CNS) tissue from larvae with pan-neuronal TFAM overexpression, Pnt knock-down, Aop knock-down, or TFAM overexpression combined with Pnt or Aop knock-down. The expression of 606 and 519 genes are significantly altered by knock-down of Pnt and Aop respectively (Fig 7A, S7 Table). 189 genes were regulated by both Pnt and Aop knock-down and the expression of these genes is strongly positively correlated (Fig 7B, S8 Table). These transcriptomic data support our epistasis analysis (S6N and S6O Fig) and are consistent with Pnt and Yan both acting as positive regulators of Ras-ERK-ETS signaling in neurons.

The expression of 494 genes were significantly altered in control versus TFAM overexpression conditions, 671 genes in control versus TFAM overexpression combined with Pnt knock-down and 560 genes in control versus TFAM overexpression combined with Aop knock-down conditions (Fig 7C, S7 Table). Around a third of these genes are commonly mis-regulated between these conditions and the expression of these common genes is very strongly positively correlated (Fig 7D and 7E, S9 and S10 Tables). Therefore, the expression of around a third of genes mis-regulated by TFAM overexpression are unchanged by Pnt or Aop knock-down. These data show that Pnt or Aop knock-down does not enhance the transcriptional changes caused by mitochondrial retrograde signaling.

To understand how Pnt and Aop knock-down modify the mitochondrial retrograde response we directly compared the TFAM overexpression transcriptome to TFAM overexpression combined with Pnt or Aop knock-down (S7 Table). This comparison shows that in combination with TFAM overexpression, Pnt knock-down significantly alters the expression of 424 genes and Aop knock-down the expression of genes 314, compared to TFAM overexpression alone (S7 Table). Of these, 82 genes (TFAM o/e vs TFAM o/e, Pnt RNAi) and 78 genes (TFAM o/e vs TFAM o/e, Aop RNAi) are also mis-regulated in control versus TFAM overexpression conditions (S11 and S12 Tables). Interestingly, the expression levels of these genes are strongly negatively correlated (Fig 7F and 7G). Around half of these genes are mis-regulated by both Pnt and Aop knock-down (S13 Table). Therefore, Pnt or Aop knock-down reverses the expression of a minor subset of genes that are mis-regulated in response to mitochondrial dysfunction. The enriched GO terms for these genes shows that Pnt or Aop knock-down reverses the expression of a number of functional classes, including transcriptional regulation and transmembrane helix, which may contribute to improved neuronal function (S14 Table). Alternatively, Pnt and Aop knock-down may alleviate the effects of mitochondrial dysfunction through the action of the genes that are regulated independent of retrograde signalling. To test this directly we used RNAi to knock-down Hsc70-2. *Hsc70-2* encodes a chaperone of the heat shock 70 family and is the most strongly upregulated gene in TFAM overexpression conditions (S7 Table). Knock-down of either Pnt or Aop dramatically reduces the retrograde induced upregulation of *Hsc70-2* expression (S11 and S12 Tables). Knock-down of Hsc70-2 in motor neurons suppresses the climbing and wing inflation phenotypes caused by TFAM overexpression (Fig 7H and 7I). These results are consistent with a mechanism whereby inhibition of Ras-ERK-ETS signaling alleviates the damaging effects of neuronal mitochondrial dysfunction by reversing the expression of a specific subset of genes within the mitochondrial retrograde transcriptome.

## Discussion

Fluctuations in mitochondrial activity and function occur during the cell cycle, throughout development and in disease states. Changes in mitochondrial function affect the cell at multiple levels, but the cellular response to these homeostatic changes is very poorly understood. We have devised a method to identify genes potentially involved in mitochondrial retrograde signaling in vivo. The number and function of the genes identified suggests an extensive and orchestrated cellular response to mitochondrial dysfunction. Inhibition of one of the pathways identified in the screen, Ras-ERK-ETS signaling, also alleviates the effects of mitochondrial dysfunction in the *Drosophila* nervous system. Targeting Ras-ERK-ETS signaling also improves function in *Drosophila* models of Leigh syndrome and Parkinson’s. Inhibition of Ras-ERK-ETS signaling partially reverses the mitochondrial retrograde transcriptional response, evidence that retrograde signaling contributes to neuronal dysfunction.

Proteomic and genetic methods have been highly successful in characterising the complement of proteins that make up the mitochondrion [42–44]. Human mitochondria consist of around 1158 proteins, only 13 of which are encoded by the mitochondrial genome [42]. However, mitochondria do not function in isolation and participate in a variety of cellular functions, acting within a homeostatic network that responds to changes in the cellular environment [22]. We have developed a sensitised phenotypic assay in the wing to identify genes involved in the cellular response to changes in mitochondrial activity. Using this for a genetic screen we identified 30 modifier genes, the majority of which enhance the wing phenotype. It is possible that screening in this way could identify components of the OXPHOS complexes, or other core mitochondrial proteins. However, knock-down of such genes on their own in the wing is likely to (and in our experience does) cause a wing phenotype, and so these genes would be excluded from the screen. We did identify several genes encoding cytosolic metabolic proteins (*Pgk*, *Adk1*, *Pgi*) as enhancers of mitochondrial dysfunction, which did not cause a phenotype when knocked-down by themselves but enhanced the MitoMod phenotype (S3 Table). However, a key point of this study is that many of the genes identified have roles in signal transduction and regulation of gene expression, strongly suggesting that mitochondrial dysfunction modulates the activity of a variety of cell signaling pathways.

The ETS domain transcription factor Aop was identified as a suppressor in the modifier screen and also suppressed neuronal mitochondrial dysfunction phenotypes. Knock-down or heterozygosity for *pnt* also rescued neuronal mitochondrial dysfunction phenotypes. These results are surprising, as Aop and Pnt generally act antagonistically to each other, with Aop acting as an inhibitor of Ras-ERK signaling and Pnt as a positive effector. In the canonical model, activated ERK phosphorylates both Pnt and Aop, promoting cytosolic translocation and degradation of Aop, as well as enhancing the transcriptional activity of Pnt [45, 46]. In the wing Pnt and Aop have opposite effects: knock-down of Pnt (using JF02227) enhances, while knock-down of Aop suppresses the MitoMod phenotype. However, recent systems biology approaches have shown that the dynamic interplay between Aop and Pnt is more complex than previously thought and is context dependent [47–49]. Aop stability is in fact regulated differentially by Ras-ERK signaling depending on the neuronal differentiation state [49]. We find that PntP2 and Aop overexpression and expression of a constitutively active form of Ras all strongly inhibit neuronal function and act synergistically with TFAM overexpression. Knock-down of either Pnt or Aop suppress Ras-ERK pathway activation and the gene expression changes caused by Pnt or Aop knock-down in neurons strongly correlate. Moreover, reduced expression of Ras, Pnt or Aop are protective against the effects of neuronal mitochondrial dysfunction. Aop and Pnt therefore both act as positive effectors of Ras-ERK signaling in motor neurons and in the context of neuronal mitochondrial dysfunction.

Mitochondrial activity plays a key role in healthy ageing. It was recently shown that inhibition of Ras, through ubiquitous expression of dominant negative Ras, or Ras knock-down in adult flies extends lifespan [50]. In contrast to our study, expression of an activated form of Aop in the gut and fat body of adult flies extended lifespan, while knockdown of Aop in these tissues had no effect on longevity [50]. The authors did not test whether inhibition of Pnt affected lifespan, but Pnt overexpression significantly reduced the lifespan of wild-type flies. Furthermore, administration of a pharmacological agent, Trametinib, which inhibits Ras activation of ERK kinase, increased *Drosophila* lifespan. Although there may be differences in the role of Aop in neuronal mitochondrial dysfunction versus healthy ageing, this previous study and our work together point to the exciting possibility that inhibition of Ras-ERK-ETS signaling may be beneficial to both healthy ageing and human diseases associated with mitochondrial dysfunction. Our data also suggest that Ras-ERK-ETS signaling acts both cell autonomously and non-cell autonomously in response to mitochondrial dysfunction. In the developing *Drosophila* eye, Ras-ERK signaling determines cell autonomous photoreceptor cell fate, acting downstream of the EGF receptor [51]. The secretion of Spitz, the ligand for the EGFR, from adjacent cells is also regulated by the Ras-ERK pathway [52]. Ras-ERK signaling thus acts both cell autonomously and non-cell autonomously to control photoreceptor differentiation. Mitochondrial signaling in *Drosophila* has previously been shown to act through non-autonomous systemic negative regulation of insulin signaling [53]. The mechanism of regulation of Ras-ERK-ETS in ageing is not known, nor do we know how mitochondrial dysfunction activates this pathway in neurons. A variety of mitochondrial retrograde signals have been identified, including ROS, Ca^2+^, AMP, nicotinamide adenine dinucleotide (NAD+) and acetyl coenzyme A [30]. Future studies will identify the factor(s) that mediate mitochondrial retrograde signaling in the nervous system.

Large scale alterations in transcription in response to mitochondrial dysfunction have been observed in a wide range of cell types [23]. However, whether these altered transcriptomes have a functional consequence is not clear. Inhibition of Ras-ERK-ETS signaling restores neuronal function and pre-synaptic active zones in our models but does not appear to affect the primary mitochondrial defect. This is consistent with our transcriptomic data, which do not show large scale alterations in the expression of mitochondrial genes with Aop or Pnt Knock-down. We suggest that the beneficial effects of Ras-ERK-ETS pathway inhibition on mitochondrial dysfunction result from transcriptional reprogramming that leads to improvement in pre-synaptic structure and function.

Reduced expression of Aop or Pnt, transcriptional targets of Ras-ERK signaling, alleviate the effects of neuronal mitochondrial dysfunction. We exploited this finding to test how the transcriptome is affected by Aop and Pnt knock-down in the background of mitochondrial dysfunction. Surprisingly, a significant number of the mitochondrial retrograde transcriptional changes are reversed by Pnt or Aop knock-down. This finding suggests that the transcriptional mis-regulation activated by mitochondrial retrograde signaling at least partly contributes to neuronal dysfunction. In support of this idea, we find that knock-down of Hsc70-2, a retrograde response gene whose expression is reduced by Aop and Pnt knock-down, alleviates neuronal mitochondrial dysfunction phenotypes. Future analyses of these retrograde response genes will help elucidate the cellular mechanisms that contribute to neuronal dysfunction.

Individual mitochondrial diseases are rare, but in total affect up to 1 in 4300 in the population [7]. The nervous system is frequently affected by mitochondrial mutations, resulting in a wide range of clinical outcomes including ataxia, epilepsy, neuropathy and deafness [54]. Treatments for mitochondrial diseases are limited and mostly symptomatic. Manipulation of the response to mitochondrial dysfunction in neurons may provide a new potentially curative strategy for mitochondrial diseases. Ras-ERK signaling has key roles in synaptic plasticity, learning and memory [55]. However, Ras-ERK-ETS signaling has not previously been identified as potential therapeutic target for mitochondrial disease. Determining how the transcriptional targets of Ras-ERK-ETS signaling contribute to neuronal dysfunction will provide important new insight into mitochondrial diseases such as Leigh syndrome.

## Materials and methods

### Fly strains and growth conditions

Flies were maintained on standard yeast, glucose, cornmeal, agar food at 25°C in a 12 hour light/dark cycle unless stated otherwise. For imaging experiments, except AT[NL] and AT[RK] experiments, embryos were laid over a 24 hour period at 25°C, incubated for a further 24 hours at 25°C, then incubated at 29°C for three days prior to analysis. For AT[NL] and AT[RK] experiments embryos were laid at 25°C and larvae maintained at 25°C until dissection.

Fly stocks were *UAS-TFAM3M* [36], which was used for all TFAM overexpression experiments except where the weaker *UAS-TFAM10M* [36] is stated, *park*^*25*^ [40], *UAS-Surf1*^*23*.*4*^ RNAi [41], *Ras85D*^*ΔC40B*^ [56] and *UAS-mito-roGFP2-Grx1* [57]. *UAS-ATeam1*.*03NL* (AT[NL]) and *UAS-ATeam1*.*03RK* (AT[RK]) flies [39] were from the Kyoto Stock Center (DGRC). The following fly stocks were from the Bloomington Stock Center: DJ-1α, DJ-1β, Lrrk, parkin and Pink1 RNAi and overexpression lines (details in S1 Table), *w*^*1118*^, *FRT82B*, *TFAM*^*c01716*^, *Da-Gal4*, *nSyb-Gal4*, *UAS-mitoGFP*, *MS1096-Gal4*, *OK371-Gal4*, *D42-Gal4*, *UAS-CD8GFP*, *UAS-Aop*, *UAS-PntP2*, *pnt*^*Δ88*^, *Thor-lacZ* (*Thor*^*K13517*^*)*, *tub-Gal4*, *TM6B*, *tub-Gal80*, *GMR-Gal4*, *UAS-Ras*^*V12*^, *TFAM*^*JF02307*^ and *TFAM*^*HMC04965*^ RNAi lines, *Dsor*^*HMS00037*^ and *Dsor*^*JF01697*^ RNAi lines, *Rl*^*JF1080*^ and *Rl*^*HMS00173*^ RNAi lines. The TFAM RNAi line (NIG, 4217R-1), used in the MitoMod stock (*MS1096-Gal4*; TFAM RNAi, *TFAM*^*c01716*^*/ TM6B*, *tub-Gal80)*, was from the NIG-Fly Stock Center, Japan. RNAi stocks used in the genetic screen were from the Bloomington Stock Center, the Vienna Drosophila Resource Center [58] and the NIG-Fly Stock Center, Japan and are listed in S2, S5 and S6 Tables. *park*^*25*^, *Ras85D*^*ΔC40B*^ and *park*^*25*^, *pnt*^*Δ88*^ lines used in Fig 6 were generated by recombination. Mosaic analysis with a repressible cell marker (MARCM) analysis of *FRT82B*, *TFAM*^*c01716*^ was performed using *y*, *w*,*hs-flp;tub-Gal4*,*UAS-mCD8GFP;FRT82B*,*tub-Gal80* flies as in Avet-Rochex et. al. [59]. Gene names are according to Flybase [60].

### Genetic screen

Virgin female flies carrying the MitoMod genotype balanced with *TM6B*,*tub-Gal80* (*MS1096-Gal4; TFAM RNAi*,*TFAM*^*c01716*^*/TM6B*,*tub-Gal80)* were crossed to males carrying RNAi transgenes. 1–2 days after eclosion of the progeny wings were observed and scored in males. RNAi lines were only classed as enhancers if most flies had a ≥90ᵒ wing curve. RNAi lines were classed as suppressors if most flies had a <45ᵒ wing curve (S2 Table). Crosses from RNAi lines that enhanced or suppressed the MitoMod wing phenotype were repeated to confirm the result. To exclude RNAi lines that cause a phenotype by themselves, *MS1096-Gal4* virgin females were crossed to all RNAi lines. If the progeny of this cross had a wing phenotype then the RNAi line was omitted from the MitoMod screen (S2 Table). Genes for all interacting RNAi lines were tested with independent RNAi lines, where available (S2 Table), and only classed as positive hits if the phenotype was replicated by the independent RNAi (S5 and S6 Tables).

RNAi lines were selected using gene expression data available on FlyAtlas, to select genes that are expressed more strongly in the brain than in the whole body [61]. RNAi lines for *Drosophila* chromatin remodelling genes were also used [62]. GO analysis of genes identified in the screen was performed using the Panther Classification System [63].

### Behavioral analysis

Climbing assays were performed as previously described [36]. Males were used for all climbing assays, apart from experiments involving knock-down of Surf1, where females were used for all genotypes.

To quantify wing inflation, flies were transferred into a new vial after eclosion and left for at least 24 hours to allow time for normal wing inflation to occur. Numbers of flies with fully inflated, semi-inflated and uninflated wings were then recorded. All flies that eclosed from the vial were counted. Statistical analysis was performed on raw data and data displayed as a percentage.

### Immunofluorescence and imaging

Tissues were prepared, imaged and quantified as previously described [36]. Primary antibodies were *Drosophila* anti-TFAM (Abcam, 1/500), rabbit anti-Dcp1 (Cell Signaling, 1/200), mouse anti-Wingless (DSHB, 1/200), chicken anti-β galactosidase (Abcam ab9361, 1:1000), mouse anti-Aop (DSHB, 1/200), mouse anti-brp (DSHB, 1/200), rabbit anti-dpErk1/2 (Cell Signaling, 1/200), HRP-Cy3 (Stratech, 1/1000), rat anti-PntP2 (1/500) [64]. Secondary antibodies were AlexaFlour 488, AlexaFlour 594 and AlexaFlour 633 (Invitrogen, 1/1000). All images were taken using a Zeiss LSM710 confocal microscope with Zen software. Imaging of controls and experimental samples in each experiment was performed using identical confocal microscope settings. Mitochondrial number and volume were quantified using Volocity (Perkin Elmer), 15μm x 15μm (xy), 5 μm (z). dpERK expression was quantified in ImageJ using the Point and Measure tools. Dcp1 expression was quantified using Volocity (PerkinElmer) using image projections. To quantify Dcp1 expression in the wing disc the dorsal compartment was selected as the area between the wingless expressing dorsoventral boundary and the third fold in the hinge area of the wing disc. MARCM analysis was performed as in Avet-Rochex et. al. [59].

For Förster resonance energy transfer (FRET)–based ATP biosensor imaging, AT[NL] ATP biosensor expressing and AT[RK] ATP insensitive expressing control wing imaginal discs or larval CNS tissues were dissected in Schneider’s medium (Thermo Scientific) and imaged immediately at 21°C using a 458nm excitation laser and detecting emitted light between 460-499nm (CFP) and 535-650nm (FRET) using a Zeiss LSM 710 confocal microscope. For control experiments wing imaginal discs were incubated in Schneider’s medium with 100μM oligomycin (VWR)/50 mM 2-deoxyglucose (SLS) for 40 minutes at 21°C, then imaged immediately in the same medium. CFP and FRET channel signal intensity at the same three randomly selected points in each wing disc, or three cell bodies in each VNC, was determined using the ImageJ Point and Measure tools and used to calculate the FRET/CFP ratio as a measure of ATP levels [39]. Imaging using Mito-roGFP2-Grx1 was performed as described previously [36]. For DHE staining, wing discs were incubated in 2μM DHE (Cambridge Bioscience) in Schneider’s medium for 10 minutes, rinsed twice in Schneider’s medium, then fixed for 5 minutes in 4% formaldehyde/PBS, washed briefly in PBS, dissected and imaged.

### ATP luciferase assay

20 wing imaginal discs per genotype were dissected in PBS, homogenized in 100μl extraction buffer (6M guanidine chloride, 100mM TrisHCL, 4mM EDTA, pH 8.0) and incubated at 70°C for 5 mins. ATP levels were measured using the ATP Determination kit (Molecular Probes) according to the manufacturer’s instructions.

### Western blot analysis

Western blot analysis was performed as previously described [36]. Primary antibodies were diluted in TBS/0.1% Tween 20 (TBS-T) and incubated overnight at 4°C and were *Drosophila* anti-TFAM (Abcam, 1/500), mouse anti-ATP5A (Abcam, 1/5000), mouse anti-MTCO1 (Abcam, 1/1000) and rabbit anti-Actin (Cell Signaling, 1/4000). After three ten minute washes in TBS-T, the membranes were incubated for 90 minutes with fluorescently labelled secondary antibodies (anti-mouse IRdye 680 and anti-rabbit IRdye 800, LI-COR, both at 1/5000) diluted in TBS-T, then washed three times for ten minutes in TBS-T. The membranes were then scanned and analysed using an Odyssey infrared scanner (LI-COR). Odyssey infrared imaging systems application software version 3.0.25 was used to quantify the intensity of the bands on the blots. Normalised expression level was calculated by determining the band intensity relative to Actin.

### Quantitative PCR

qRT-PCR and qPCR of mtDNA were performed as previously described [36]. Primers used for qRT-PCR were ThorFwd: CGAGGTGTACTCCTCGACGC and ThorRvs: GAGCCACGGAGATTCTTCATGA; Hsp22Fwd: AGCGTTGTCCTGGTGGAG and Hsp22Rvs: GAGCTATAGCCACCTTGTTCG; Rpl4Fwd: TCCACCTTGAAGAAGGGCTA and Rpl4Rvs: TTGCGGATCTCCTCAGACTT.

### Microarray experiments and analysis

CNS tissue from 20 late third instar larvae per genotype were dissected in cold PBS and transferred directly into 100μl lysis buffer containing β-mercaptoethanol (Absolutely RNA Microprep kit, Agilent Technologies). The lysis buffer was kept on ice while all the brains were dissected. RNA was prepared following manufacturer’s instructions, including DNase treatment and stored at -80°C. Samples were prepared in triplicate.

RNA was measured for quantity and integrity on an RNA Pico Chip (Agilent Technologies). 10ng of RNA per genotype was converted into labelled cDNA with the Nugen Ovation System V2 (NuGEN Technologies Inc.). 7mg of labelled cDNA was hybridised to Affymetrix *Drosophila* genome v2 GeneChips for 20 hours at 45°C. They were then washed, stained (GeneChip Fluidics Station 450) and scanned (GeneChip Scanner 3000 7G) according to the manufacturer’s instructions (Nugen Technologies Inc & Affymetrix).

Microarray data was processed using the MAS5.0 algorithm using the Transcriptome Analysis Console (ThermoFisher). Means were calculated using Tukey's Bi-weight average algorithm and differential expression between groups was calculated using un-paired one way analysis of variance (ANOVA). A statistical cutoff of p<0.05 and a fold change cutoff of ±1.5 fold were used. Correlations between datasets were analysed using GraphPad Prism (GraphPad Software Inc.). The data discussed in this publication have been deposited in NCBI's Gene Expression Omnibus and are accessible through GEO Series accession number GSE114054. GO analysis was performed using DAVID (the database for annotation, visualization and integrated discovery) bioinformatics resources [65].

### Statistical analyses

GraphPad Prism (GraphPad Software Inc.) was used to create graphs and for statistical analysis. Data with a p-value less than or equal to 0.05 was considered significant. Comparisons of two samples of continuous data were analysed with an unpaired, two-tailed student’s t-test, where appropriate. Data were analysed for normality using the D’Agostino & Pearson omnibus normality test. Data that did not pass the normality test were analysed with the Mann Whitney test. Variance of the samples was assessed with an F test. If the variances of the two samples were significantly different then the Welch’s correction was applied to the t-test. In order to compare more than two samples of continuous data, one-way ANOVA was used with Tukey’s post hoc test. If data did not pass the D’Agostino & Pearson omnibus normality test, the Kruskal-Wallis, followed by Dunn’s post hoc test were utilised. Categorical data were analysed using chi-squared.

## Supporting information

S1 Fig TFAM knockdown causes loss of mtDNA and mitochondrial gene expression. (A) qPCR of mtDNA copy number from late third instar larvae with ubiquitous knock-down of TFAM (4217R-1) using tub-Gal4. Controls are tub-Gal4 hemizygotes. (B) Western analysis of coxI, ATP synthase α and TFAM expression from late third instar larvae control (tub-Gal4/+, lanes 1–3), or with ubiquitous knock-down of TFAM using tub-Gal4 (lanes 4–6). (C-E) Quantification of coxI, ATP synthase α and TFAM expression. (F,G) TFAM staining in wing imaginal discs with TFAM knock-down (4217R-1) (F) or TFAM overexpression (G) using MS1096-Gal4. Arrows mark the dorso-ventral compartment boundary (dorsal is up). Scale bar: 10μm. (H) A MARCM clone in the late third instar larval wing imaginal disc showing that cells that are homozygous of TFAM^c01716^ (expressing GFP, green) have strongly reduced levels of TFAM expression (red in H, white in H’). (I-L) TFAM overexpression (using TFAM10M grown at 18°C to reduce Gal4 activity), or knock-down of TFAM using two independent RNAi lines (TFAM^JF02307^ and TFAM^HMC04965^) in the wing using MS1096-Gal4 cause a curved wing phenotype. (M-P) The curved wing phenotype caused by knock-down of TFAM using MS1096-Gal4 and heterozygosity for TFAM^c01716^ (M,O) is almost completely rescued by co-expression of TFAM in both males (N) and females (P).
(TIF)

S2 Fig TFAM knock-down and overexpression alter mitochondrial morphology but do not affect ATP levels in the developing wing. (A-C) The FRET/CFP fluorescence emission ratio of the AT[NL] FRET–based ATP biosensor expressed in the wing imaginal disc using MS1096-Gal4 is decreased when the tissue is incubated with oligomycin (OM)/2-deoxyglucose. (D-F) The FRET/CFP fluorescence emission ratio of the AT[RK] control protein, which does not bind ATP, is unchanged when the tissue is incubated with oligomycin (OM). (G-J) Knock-down (4217R-1) or overexpression of TFAM do not alter the FRET/CFP fluorescence emission ratio of the AT[NL] FRET–based ATP biosensor in the wing disc. Images show a merge of the CFP (green) and FRET (red) channels. (K) ATP luciferase assay of wing discs with TFAM RNAi and overexpression using MS1096-Gal4. (L-N) Ratio images show no change in mitochondrial glutathione redox potential reporter mito-roGFP2-Grx1 fluorescence after excitation at 405nm (red) and 488nm (green) in wing discs with TFAM knock-down and overexpression using MS1096-Gal4. (O) Quantification of mito-roGFP2-Grx1 fluorescence ratio. (P-R) DHE staining in MS1096-Gal4 hemizygous control (P), TFAM knock-down (Q) and overexpression (R) wing discs. (S) Quantification of DHE staining in the dorsal compartment of the wing disc. (T-V) Mitochondrial morphology with TFAM knock-down (U) and TFAM overexpression (V) in the wing imaginal disc using MS1096-Gal4, compared to control (T). Mito-GFP is used to label mitochondria. Scale bar: 10 μm. (W,X) Quantification of mitochondrial number (W) and volume (X) in wing imaginal discs. (Y,Z) qRT-PCR of Thor (Y) and Hsp22 (Z) mRNA expression in wing imaginal discs with TFAM knock-down and TFAM overexpression using MS1096-Gal4. Data are represented as mean +/- SEM, n.s. not significant, *p≤0.05, **≤0.01, ***p≤0.001, a.u. arbitrary units.
(TIF)

S3 Fig Modulation of genes associated with Parkinson’s disease enhance the MitoMod wing phenotype. (A) MS1096-Gal4, + control male. (B) Male progeny from MitoMod fly crossed to w^1118^ showing the 45° curve at the wing tip. (C-H) Male progeny from crosses of MitoMod with DJ-1α RNAi (HMJ21180) (C), DJ-1α overexpression (D), DJ-1β RNAi (HMS01915) (E), DJ-1β overexpression (F), Lrrk RNAi (HMS00456) (G), Pink1 overexpression (H).
(TIF)

S4 Fig Knock-down of Aop or Ino80 abrogates the increased apoptosis phenotype caused by knock-down of TFAM. (A) A wing disc from a MS1096-Gal4, + larva stained for Dcp1 expression and DAPI. (B,C) Wing discs from larvae with knock-down of Ino80 and Aop using MS1096-Gal4. (D) A wing disc from the progeny of MitoMod crossed to w^1118^. (E,F) Wing discs from the progeny of MitoMod crossed to Ino80 RNAi (E) and Aop RNAi (F). (G,H) Quantification of Dcp1 expression. Dcp1 expression is shown in green in (A)-(F) and white in (A’)-(F’) and DAPI staining shown in blue. Dotted line marks the dorso-ventral compartment boundary (dorsal is bottom left). (I) Quantification of Dcp1 expression in MitoMod wing discs combined with knock-down of Chrac-14, Ing3 and MTA1-like. (J) Quantification of Dcp1 expression in wing discs overexpressing TFAM combined with knock-down of Ino80 or Aop. Data are represented as mean +/- SEM, n.s. not significant, *p≤0.05, ***p≤0.001.
(TIF)

S5 Fig Knock-down of TFAM in motor neurons causes weak mitochondrial loss and climbing phenotypes. (A-C) Segment A3, muscle 4 NMJ in late third instar larvae from control (A), with TFAM RNAi (4217R-1) (B), or TFAM overexpression (C) in motor neurons using OK371-Gal4. Motor neuron specific expression of mitoGFP (green in (A-C) and white in (A’-C’)) was used to visualise mitochondria and staining for horse radish peroxidase (red) to visualise neuronal membranes. (D,E) Quantification of mitochondrial number (D) and volume (E). (F,G) Knock-down of TFAM (4217R-1) using either OK371-Gal4 (F) or D42-Gal4 (G) causes reduced climbing ability in adults, but this phenotype is weaker than with TFAM overexpression. (H,I) An eye imaginal disc from a control GMR-Gal4/+ larva (H), or a larva expressing Aop RNAi (3166R-1) using GMR-Gal4 (I), which shows almost complete loss of Aop expression in photoreceptor neurons posterior to the morphogenetic furrow (arrow), where GMR-Gal4 is expressed (posterior is to the right). (J,K) Adult eyes from GMR-Gal4/+ control (J), or GMR-Gal4/Aop RNAi (3166R-1) (K) flies showing a rough eye phenotype caused by Aop knock-down. (L-N) Overexpression of TFAM with D42-Gal4 causes wings to either inflate normally (L), semi-inflate (M) or fail to inflate (N) in around 50% of flies. (O-R) Knock-down of Dsor using independent RNAi lines (HMS00037 and JF01697) suppresses the climbing (O,Q) and wing inflation phenotypes (P,R) caused by TFAM overexpression with D42-Gal4. (S-V) Knock-down of Rl using independent RNAi lines (JF1080 and HMS00173) suppresses the climbing (S,U) and wing inflation phenotypes (T,V) caused by TFAM overexpression with D42-Gal4. The numbers of flies counted for each genotype are shown in white. Data are represented as mean +/- SEM, n.s. not significant, *p≤0.05, **≤0.01, ***p≤0.001. Controls are Gal4 hemizygotes.
(TIF)

S6 Fig TFAM overexpression climbing and wing inflation phenotypes are suppressed by heterozygosity for Pnt. (A,B) An eye imaginal disc from a control GMR-Gal4/+ larva (A), or a larva expressing Pnt RNAi (JF02227) using GMR-Gal4 (B), which shows almost complete loss of PntP2 expression in photoreceptor neurons posterior to the morphogenetic furrow (arrow). Posterior is to the right. (C,D) Adult eyes from GMR-Gal4/+ control (C), or GMR-Gal4/Pnt RNAi (JF02227) (D) flies showing a rough eye phenotype caused by Pnt knock-down. (E) The reduced climbing ability of flies with TFAM overexpression using D42-Gal4, is suppressed in a pnt^Δ88^ heterozygous background. (F) The wing inflation phenotype caused by overexpression of TFAM with D42-Gal4 is suppressed in a pnt^Δ88^ heterozygous background. The numbers of flies counted for each genotype are shown in white. (G,H) Knock-down of Pnt combined with heterozygosity for Ras85D suppresses the climbing (G) and wing inflation phenotypes (H) caused by TFAM overexpression with D42-Gal4 compared to either condition alone. (I,J) Knock-down of Aop combined with heterozygosity for Ras85D does not affect the climbing (I) and wing inflation phenotypes (J) caused by TFAM overexpression with D42-Gal4 compared to either condition alone. (K,L) Knock-down of Pnt and Aop together does not affect the climbing (K), but suppresses the wing inflation phenotype (L) caused by TFAM overexpression with D42-Gal4 compared to Aop knock-down alone. (M) Overexpression of PntP2 in motor neurons with D42-Gal4 causes reduced climbing and is lethal when combined with TFAM overexpression. (N,O) Knock-down of Aop (N) or Pnt (O) rescues the lethality caused by expression of Ras85D^V12^ with OK371-Gal4. Controls are D42-Gal4 hemizygotes. Data are represented as mean +/- SEM, n.s. not significant, *p≤0.05, ** p≤0.01 ***p≤0.001. (P,Q) Mosaic analysis with a repressible cell marker (MARCM) control (P) or TFAM overexpression (Q) clones (green) stained for dpERK expression (red).
(TIF)

S7 Fig Knock-down of Aop does not affect the synaptic mitochondrial loss caused by mitochondrial dysfunction. (A-C) Overexpression of TFAM does not alter the FRET/CFP fluorescence emission ratio of the AT[NL] FRET–based ATP biosensor expressed in motor neurons with OK371-Gal4. Images show a merge of the CFP (green) and FRET (red) channels. Data are represented as mean +/- SEM, a.u. arbitrary units. (D,E) Quantification of mitochondrial number (D) and volume (E) from segment A3, muscle 4 NMJ in late third instar larvae from control, or with Aop RNAi (3166R-1), TFAM overexpression, or Aop RNAi (3166R-1) and TFAM overexpression together in motor neurons using OK371-Gal4. (F) Quantification of active zone number from the same genotypes as in (D,E). Controls are OK371-Gal4 hemizygotes. (G) dpERK expression is increased in the VNC by pan-neuronal knock down of Surf1 with nSyb-Gal4, compared to hemizygous nSyb-Gal4 controls. (H) dpERK expression is unchanged in the VNC in park^25^ larvae compared to w^1118^ controls. Data are represented as mean +/- SEM, n.s. not significant, *p≤0.05, ** p≤0.01, ***p≤0.001.
(TIF)

S8 Fig Knock-down of Pnt rescues the Surf1 RNAi phenotype. (A,B) Knock down of Pnt rescues the climbing (A) and wing inflation (B) phenotypes causes by knock-down of Surf1 with nSyb-Gal4. (C) The climbing defect in park^25^ male flies is not improved by ubiquitous knock-down of Aop (3166R-1) using Da-Gal4. Controls are w^1118^. Data are represented as mean +/- SEM, n.s not significant,** p≤0.01***, p≤0.001.
(TIF)

S1 Table Enhancement of the MitoMod wing phenotype by knock-down of familial Parkinson’s disease genes. (DOCX)

S2 Table RNAi lines screened in the MitoMod modifier screen. The outcome of the screen is in the Result column. Any lines that had a phenotype with MS1096-Gal4 alone were excluded from the screen, so the result reads ‘Excluded’. The confirmed column refers to confirmation of the result with an independent RNAi for the same gene, Y: confirmed, N: not confirmed, see S5 and S6 Tables for details. If the Confirmed column is blank, then an alternative RNAi was not available.
(XLSX)

S3 Table Enhancers identified in the screen with GO molecular function. (XLSX)

S4 Table Suppressor genes identified in the genetic screen with GO molecular function. (XLSX)

S5 Table Independent RNAi lines used to validate enhancers. Any lines that had a phenotype with MS1096-Gal4 alone read ‘Excluded’ in the Result column.
(XLSX)

S6 Table Independent RNAi lines used to validate suppressors. Any lines that had a phenotype with MS1096-Gal4 alone read ‘Excluded’ in the Result column.
(XLSX)

S7 Table Genes whose expression is significantly altered in the transcriptomic analyses. (XLSX)

S8 Table Genes whose expression is significantly altered in control vs Pnt RNAi and control vs Aop RNAi conditions. (XLSX)

S9 Table Genes whose expression is significantly altered in control vs TFAM overexpression and control vs TFAM overexpression combined with Pnt RNAi. (XLSX)

S10 Table Genes whose expression is significantly altered in control vs TFAM overexpression and control vs TFAM overexpression combined with Aop RNAi. (XLSX)

S11 Table Genes whose expression is significantly altered in control vs TFAM overexpression and TFAM overexpression vs TFAM overexpression combined with Pnt RNAi. (XLSX)

S12 Table Genes whose expression is significantly altered in control vs TFAM overexpression and TFAM overexpression vs TFAM overexpression combined with Aop RNAi. (XLSX)

S13 Table Genes whose expression is significantly altered in control vs TFAM overexpression and TFAM overexpression vs TFAM overexpression combined with Pnt RNAi and TFAM overexpression vs TFAM overexpression combined with Aop RNAi. (XLSX)

S14 Table Functional annotation of GO classes of genes whose expression is significantly altered in TFAM overexpression versus TFAM overexpression combined with Pnt knock-down or TFAM overexpression combined with Aop knock-down conditions. (XLSX)
