# Supplementary figures and images for: Ras-ERK-ETS inhibition alleviates neuronal mitochondrial dysfunction by reprogramming mitochondrial retrograde signaling
Source: PLoS Genet. 2018 Jul 30;14(7):e1007567. doi: 10.1371/journal.pgen.1007567 (PMC6085068; doi:10.1371/journal.pgen.1007567)

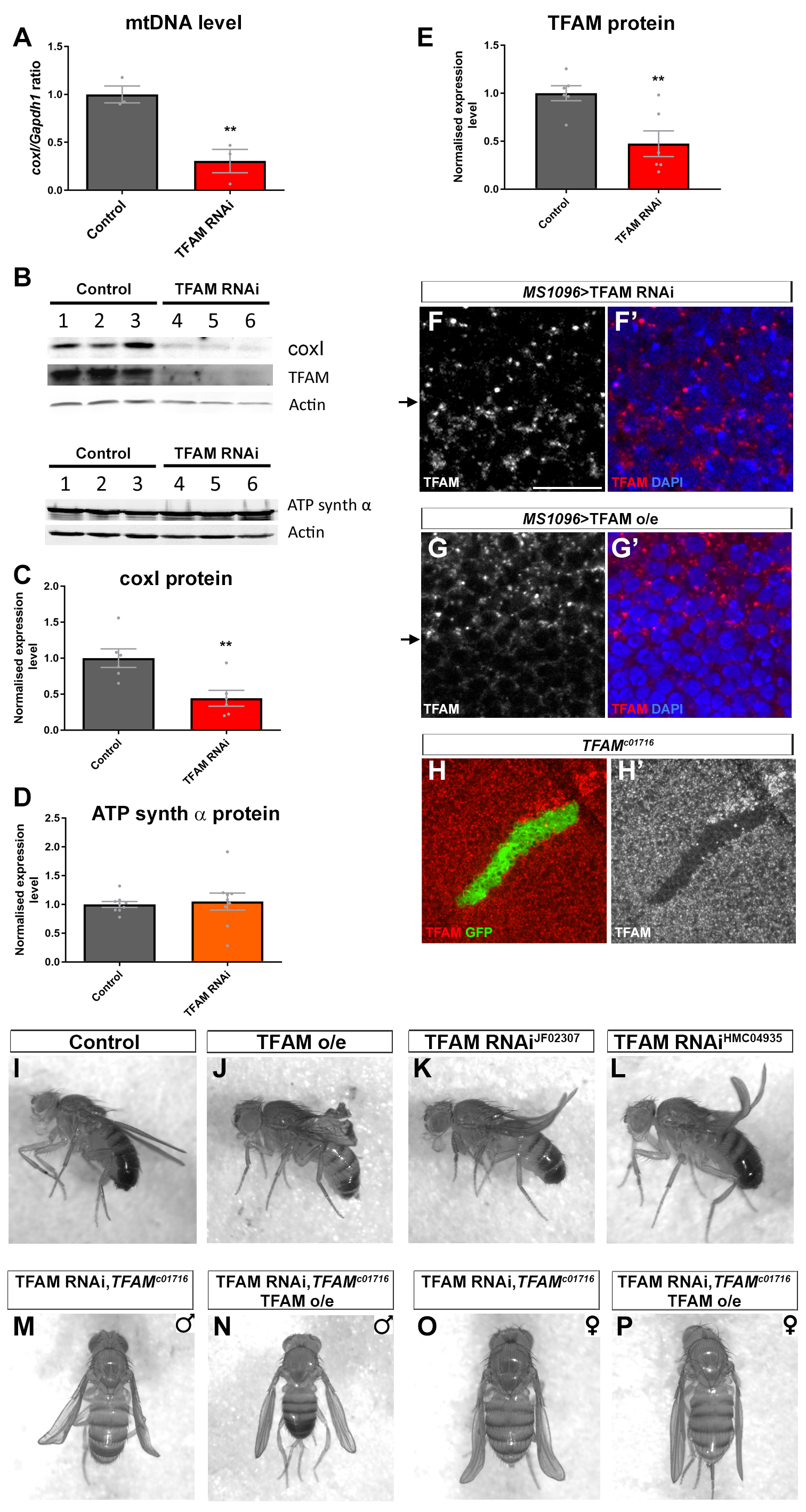

Supplement: S1 Fig — (A) qPCR of mtDNA copy number from late third instar larvae with ubiquitous knock-down of TFAM (4217R-1) using tub-Gal4. Controls are tub-Gal4 hemizygotes. (B) Western analysis of coxI, ATP synthase α and TFAM expression from late third instar larvae control (tub-Gal4/+, lanes 1–3), or with ubiquitous knock-down of TFAM using tub-Gal4 (lanes 4–6). (C-E) Quantification of coxI, ATP synthase α and TFAM expression. (F,G) TFAM staining in wing imaginal discs with TFAM knock-down (4217R-1) (F) or TFAM overexpression (G) using MS1096-Gal4. Arrows mark the dorso-ventral compartment boundary (dorsal is up). Scale bar: 10μm. (H) A MARCM clone in the late third instar larval wing imaginal disc showing that cells that are homozygous of TFAMc01716 (expressing GFP, green) have strongly reduced levels of TFAM expression (red in H, white in H’). (I-L) TFAM overexpression (using TFAM10M grown at 18°C to reduce Gal4 activity), or knock-down of TFAM using two independent RNAi lines (TFAMJF02307 and TFAMHMC04965) in the wing using MS1096-Gal4 cause a curved wing phenotype. (M-P) The curved wing phenotype caused by knock-down of TFAM using MS1096-Gal4 and heterozygosity for TFAMc01716 (M,O) is almost completely rescued by co-expression of TFAM in both males (N) and females (P). (TIF) [file pgen.1007567.s001.tif]

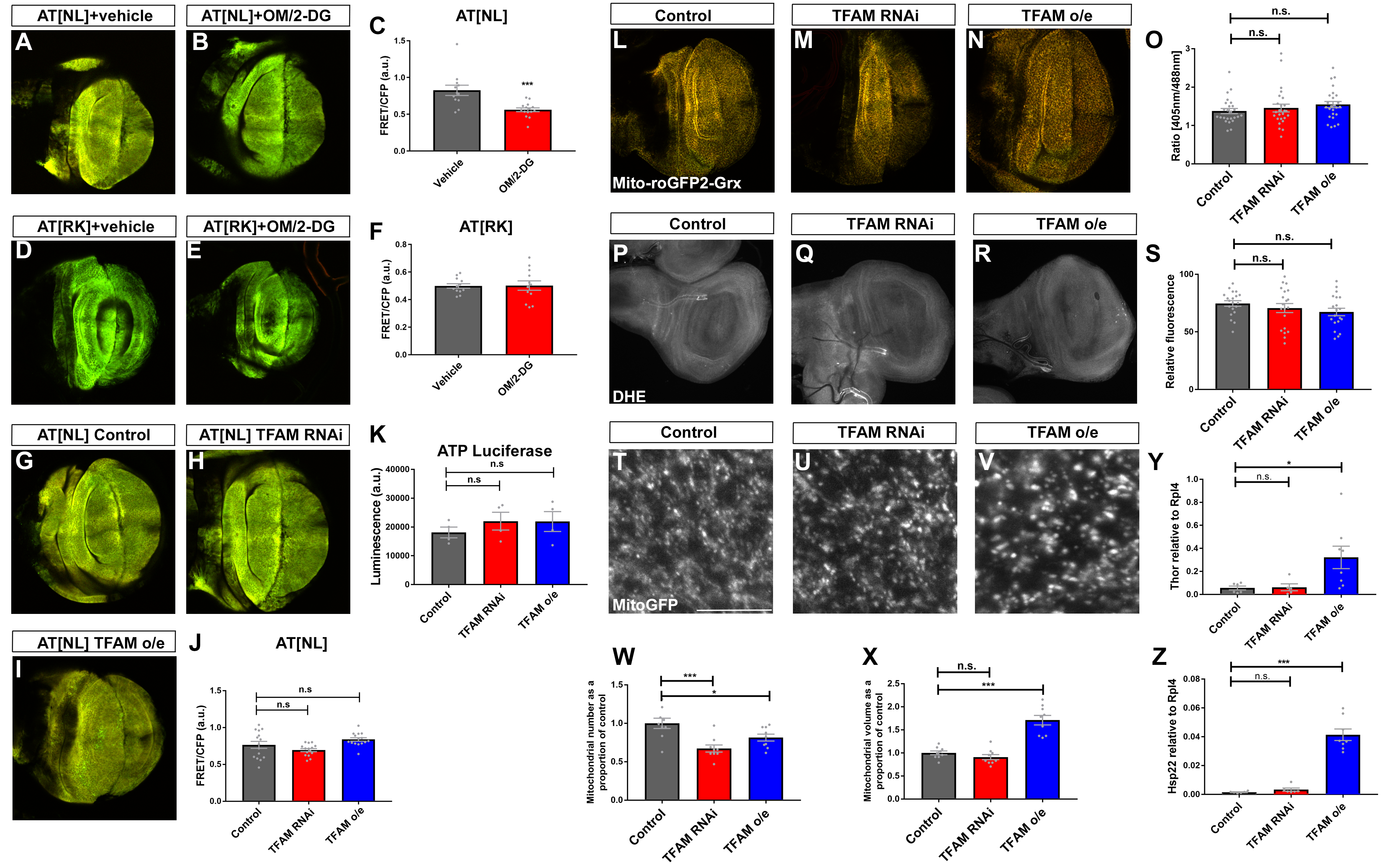

Supplement: S2 Fig — (A-C) The FRET/CFP fluorescence emission ratio of the AT[NL] FRET–based ATP biosensor expressed in the wing imaginal disc using MS1096-Gal4 is decreased when the tissue is incubated with oligomycin (OM)/2-deoxyglucose. (D-F) The FRET/CFP fluorescence emission ratio of the AT[RK] control protein, which does not bind ATP, is unchanged when the tissue is incubated with oligomycin (OM). (G-J) Knock-down (4217R-1) or overexpression of TFAM do not alter the FRET/CFP fluorescence emission ratio of the AT[NL] FRET–based ATP biosensor in the wing disc. Images show a merge of the CFP (green) and FRET (red) channels. (K) ATP luciferase assay of wing discs with TFAM RNAi and overexpression using MS1096-Gal4. (L-N) Ratio images show no change in mitochondrial glutathione redox potential reporter mito-roGFP2-Grx1 fluorescence after excitation at 405nm (red) and 488nm (green) in wing discs with TFAM knock-down and overexpression using MS1096-Gal4. (O) Quantification of mito-roGFP2-Grx1 fluorescence ratio. (P-R) DHE staining in MS1096-Gal4 hemizygous control (P), TFAM knock-down (Q) and overexpression (R) wing discs. (S) Quantification of DHE staining in the dorsal compartment of the wing disc. (T-V) Mitochondrial morphology with TFAM knock-down (U) and TFAM overexpression (V) in the wing imaginal disc using MS1096-Gal4, compared to control (T). Mito-GFP is used to label mitochondria. Scale bar: 10 μm. (W,X) Quantification of mitochondrial number (W) and volume (X) in wing imaginal discs. (Y,Z) qRT-PCR of Thor (Y) and Hsp22 (Z) mRNA expression in wing imaginal discs with TFAM knock-down and TFAM overexpression using MS1096-Gal4. Data are represented as mean +/- SEM, n.s. not significant, *p≤0.05, **≤0.01, ***p≤0.001, a.u. arbitrary units. (TIF) [file pgen.1007567.s002.tif]

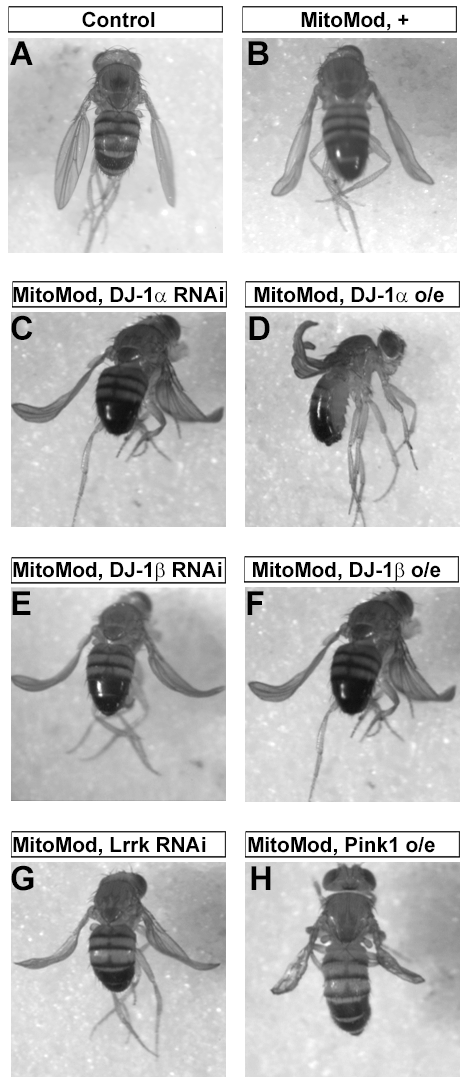

Supplement: S3 Fig — (A) MS1096-Gal4, + control male. (B) Male progeny from MitoMod fly crossed to w1118 showing the 45° curve at the wing tip. (C-H) Male progeny from crosses of MitoMod with DJ-1α RNAi (HMJ21180) (C), DJ-1α overexpression (D), DJ-1β RNAi (HMS01915) (E), DJ-1β overexpression (F), Lrrk RNAi (HMS00456) (G), Pink1 overexpression (H). (TIF) [file pgen.1007567.s003.tif]

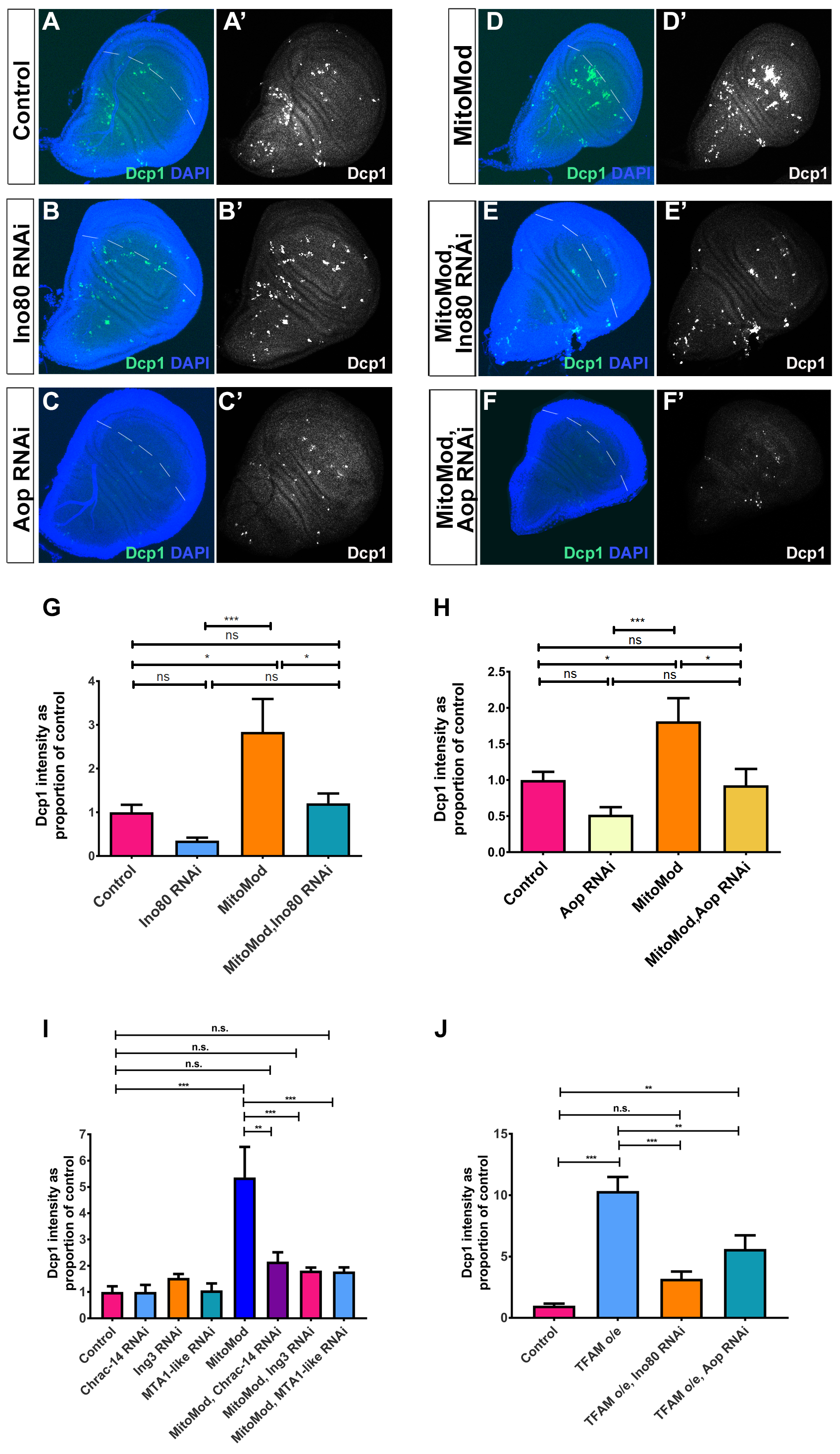

Supplement: S4 Fig — (A) A wing disc from a MS1096-Gal4, + larva stained for Dcp1 expression and DAPI. (B,C) Wing discs from larvae with knock-down of Ino80 and Aop using MS1096-Gal4. (D) A wing disc from the progeny of MitoMod crossed to w1118. (E,F) Wing discs from the progeny of MitoMod crossed to Ino80 RNAi (E) and Aop RNAi (F). (G,H) Quantification of Dcp1 expression. Dcp1 expression is shown in green in (A)-(F) and white in (A’)-(F’) and DAPI staining shown in blue. Dotted line marks the dorso-ventral compartment boundary (dorsal is bottom left). (I) Quantification of Dcp1 expression in MitoMod wing discs combined with knock-down of Chrac-14, Ing3 and MTA1-like. (J) Quantification of Dcp1 expression in wing discs overexpressing TFAM combined with knock-down of Ino80 or Aop. Data are represented as mean +/- SEM, n.s. not significant, *p≤0.05, ***p≤0.001. (TIF) [file pgen.1007567.s004.tif]

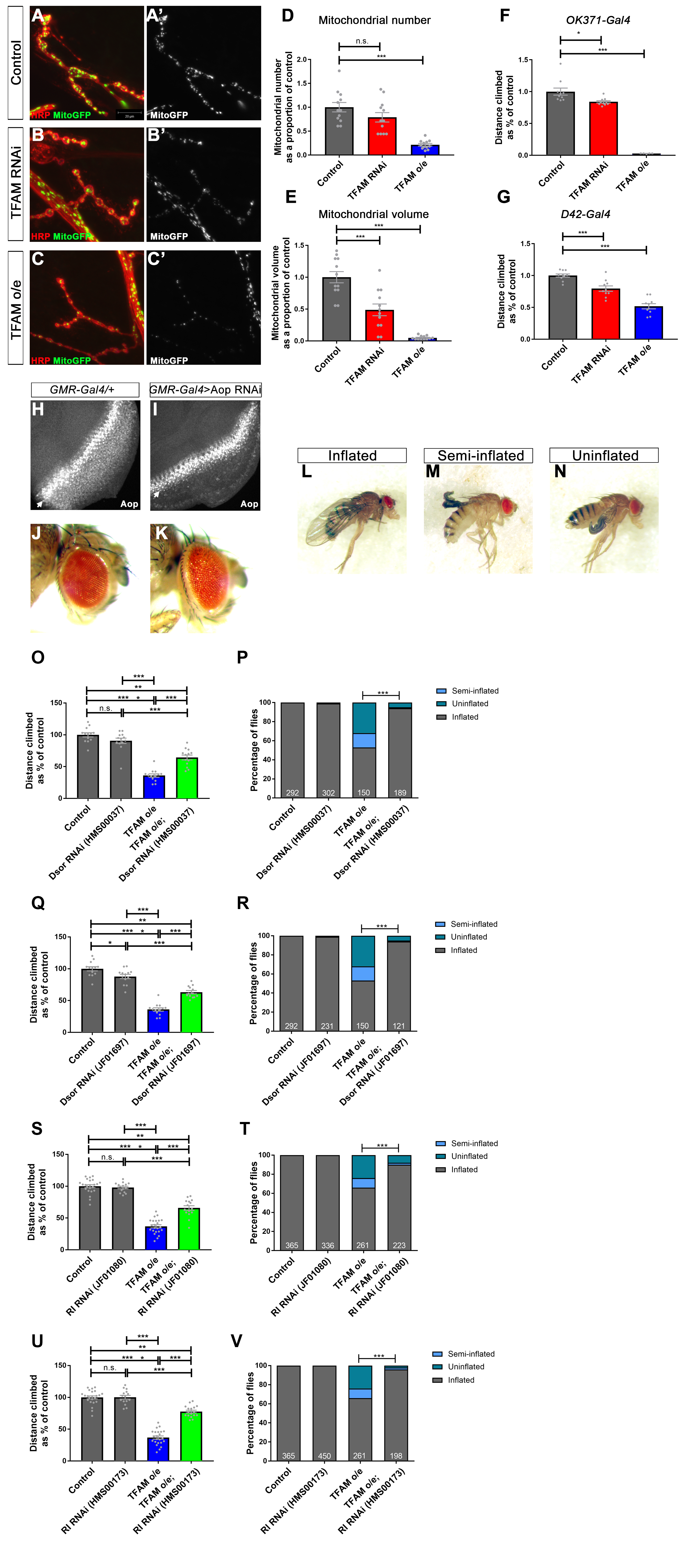

Supplement: S5 Fig — (A-C) Segment A3, muscle 4 NMJ in late third instar larvae from control (A), with TFAM RNAi (4217R-1) (B), or TFAM overexpression (C) in motor neurons using OK371-Gal4. Motor neuron specific expression of mitoGFP (green in (A-C) and white in (A’-C’)) was used to visualise mitochondria and staining for horse radish peroxidase (red) to visualise neuronal membranes. (D,E) Quantification of mitochondrial number (D) and volume (E). (F,G) Knock-down of TFAM (4217R-1) using either OK371-Gal4 (F) or D42-Gal4 (G) causes reduced climbing ability in adults, but this phenotype is weaker than with TFAM overexpression. (H,I) An eye imaginal disc from a control GMR-Gal4/+ larva (H), or a larva expressing Aop RNAi (3166R-1) using GMR-Gal4 (I), which shows almost complete loss of Aop expression in photoreceptor neurons posterior to the morphogenetic furrow (arrow), where GMR-Gal4 is expressed (posterior is to the right). (J,K) Adult eyes from GMR-Gal4/+ control (J), or GMR-Gal4/Aop RNAi (3166R-1) (K) flies showing a rough eye phenotype caused by Aop knock-down. (L-N) Overexpression of TFAM with D42-Gal4 causes wings to either inflate normally (L), semi-inflate (M) or fail to inflate (N) in around 50% of flies. (O-R) Knock-down of Dsor using independent RNAi lines (HMS00037 and JF01697) suppresses the climbing (O,Q) and wing inflation phenotypes (P,R) caused by TFAM overexpression with D42-Gal4. (S-V) Knock-down of Rl using independent RNAi lines (JF1080 and HMS00173) suppresses the climbing (S,U) and wing inflation phenotypes (T,V) caused by TFAM overexpression with D42-Gal4. The numbers of flies counted for each genotype are shown in white. Data are represented as mean +/- SEM, n.s. not significant, *p≤0.05, **≤0.01, ***p≤0.001. Controls are Gal4 hemizygotes. (TIF) [file pgen.1007567.s005.tif]

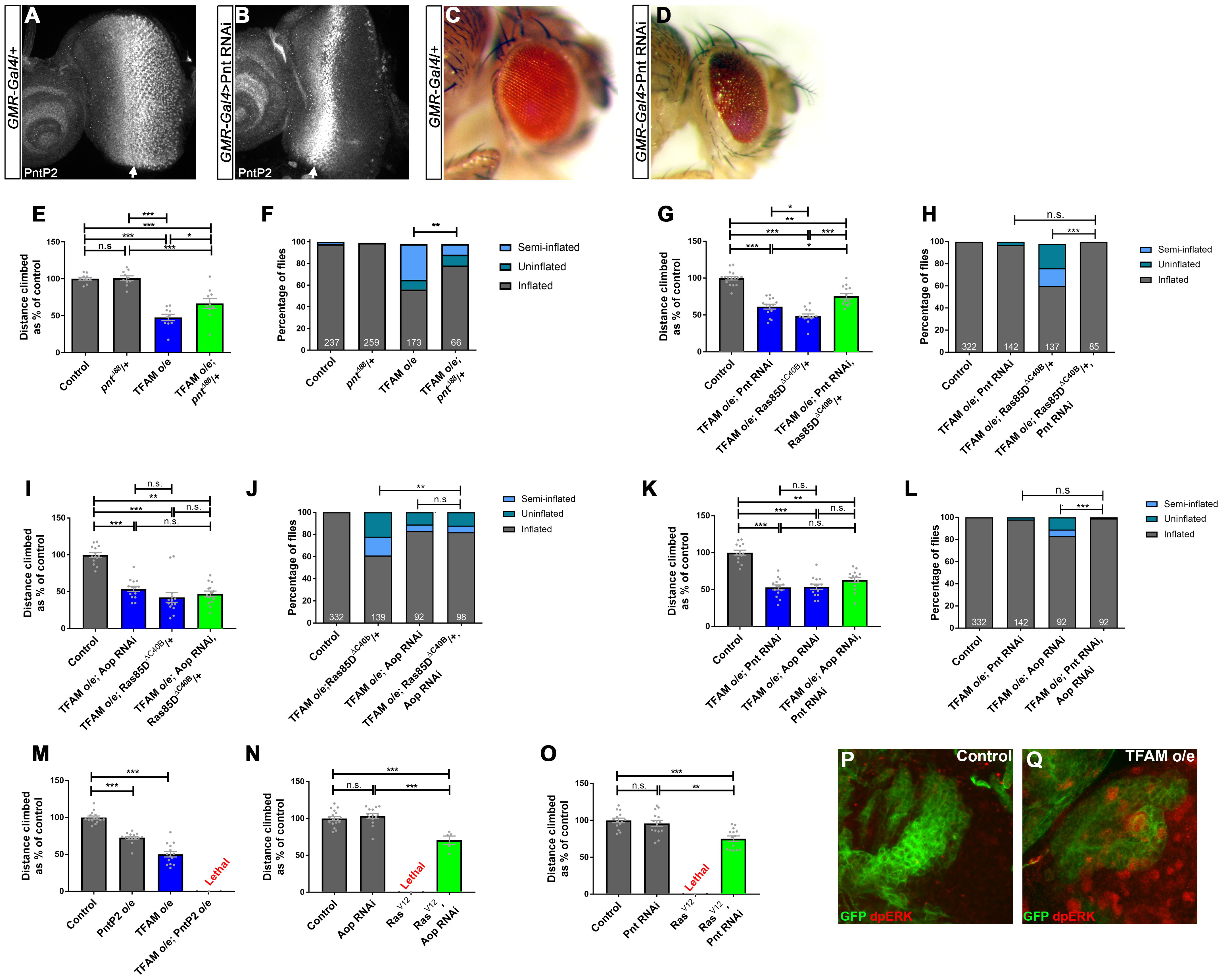

Supplement: S6 Fig — (A,B) An eye imaginal disc from a control GMR-Gal4/+ larva (A), or a larva expressing Pnt RNAi (JF02227) using GMR-Gal4 (B), which shows almost complete loss of PntP2 expression in photoreceptor neurons posterior to the morphogenetic furrow (arrow). Posterior is to the right. (C,D) Adult eyes from GMR-Gal4/+ control (C), or GMR-Gal4/Pnt RNAi (JF02227) (D) flies showing a rough eye phenotype caused by Pnt knock-down. (E) The reduced climbing ability of flies with TFAM overexpression using D42-Gal4, is suppressed in a pntΔ88 heterozygous background. (F) The wing inflation phenotype caused by overexpression of TFAM with D42-Gal4 is suppressed in a pntΔ88 heterozygous background. The numbers of flies counted for each genotype are shown in white. (G,H) Knock-down of Pnt combined with heterozygosity for Ras85D suppresses the climbing (G) and wing inflation phenotypes (H) caused by TFAM overexpression with D42-Gal4 compared to either condition alone. (I,J) Knock-down of Aop combined with heterozygosity for Ras85D does not affect the climbing (I) and wing inflation phenotypes (J) caused by TFAM overexpression with D42-Gal4 compared to either condition alone. (K,L) Knock-down of Pnt and Aop together does not affect the climbing (K), but suppresses the wing inflation phenotype (L) caused by TFAM overexpression with D42-Gal4 compared to Aop knock-down alone. (M) Overexpression of PntP2 in motor neurons with D42-Gal4 causes reduced climbing and is lethal when combined with TFAM overexpression. (N,O) Knock-down of Aop (N) or Pnt (O) rescues the lethality caused by expression of Ras85DV12 with OK371-Gal4. Controls are D42-Gal4 hemizygotes. Data are represented as mean +/- SEM, n.s. not significant, *p≤0.05, ** p≤0.01 ***p≤0.001. (P,Q) Mosaic analysis with a repressible cell marker (MARCM) control (P) or TFAM overexpression (Q) clones (green) stained for dpERK expression (red). (TIF) [file pgen.1007567.s006.tif]

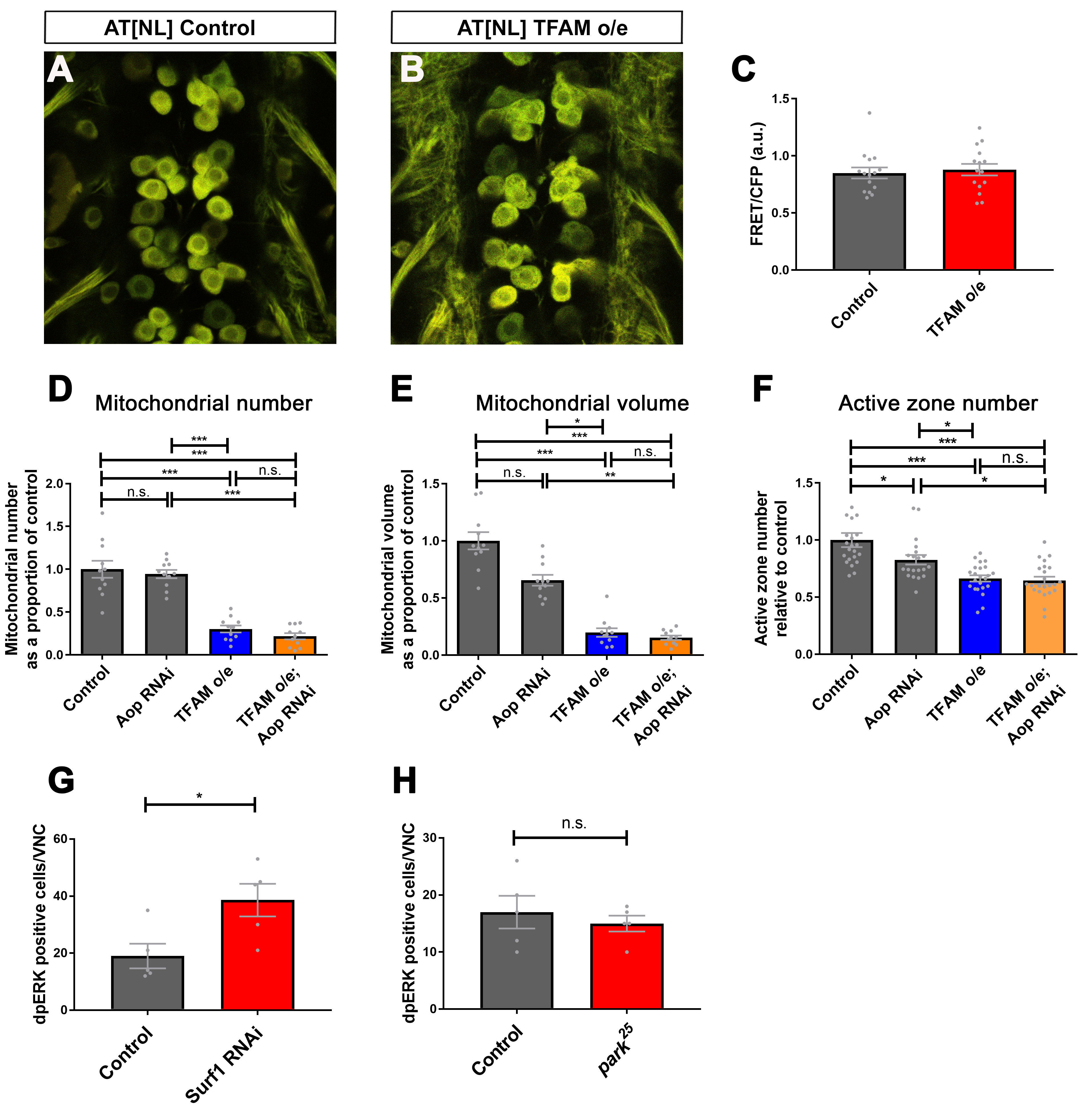

Supplement: S7 Fig — (A-C) Overexpression of TFAM does not alter the FRET/CFP fluorescence emission ratio of the AT[NL] FRET–based ATP biosensor expressed in motor neurons with OK371-Gal4. Images show a merge of the CFP (green) and FRET (red) channels. Data are represented as mean +/- SEM, a.u. arbitrary units. (D,E) Quantification of mitochondrial number (D) and volume (E) from segment A3, muscle 4 NMJ in late third instar larvae from control, or with Aop RNAi (3166R-1), TFAM overexpression, or Aop RNAi (3166R-1) and TFAM overexpression together in motor neurons using OK371-Gal4. (F) Quantification of active zone number from the same genotypes as in (D,E). Controls are OK371-Gal4 hemizygotes. (G) dpERK expression is increased in the VNC by pan-neuronal knock down of Surf1 with nSyb-Gal4, compared to hemizygous nSyb-Gal4 controls. (H) dpERK expression is unchanged in the VNC in park25 larvae compared to w1118 controls. Data are represented as mean +/- SEM, n.s. not significant, *p≤0.05, ** p≤0.01, ***p≤0.001. (TIF) [file pgen.1007567.s007.tif]

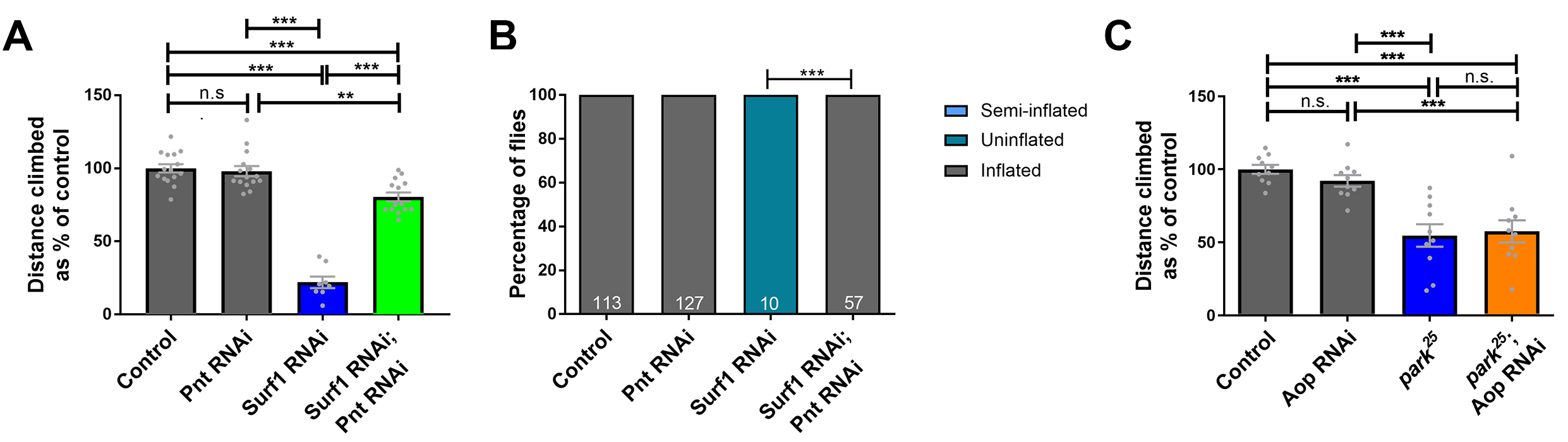

Supplement: S8 Fig — (A,B) Knock down of Pnt rescues the climbing (A) and wing inflation (B) phenotypes causes by knock-down of Surf1 with nSyb-Gal4. (C) The climbing defect in park25 male flies is not improved by ubiquitous knock-down of Aop (3166R-1) using Da-Gal4. Controls are w1118. Data are represented as mean +/- SEM, n.s not significant,** p≤0.01***, p≤0.001. (TIF) [file pgen.1007567.s008.tif]
