# Supplementary material for: Ras-ERK-ETS inhibition alleviates neuronal mitochondrial dysfunction by reprogramming mitochondrial retrograde signaling
Source: PLoS Genet. 2018 Jul 30;14(7):e1007567. doi: 10.1371/journal.pgen.1007567 (PMC6085068; doi:10.1371/journal.pgen.1007567)
Supplement: S1 Table — (DOCX) [file pgen.1007567.s009.docx]

| **Transgene** | **Type** | **Gene (CG#)** | **Bloomington stock number** | **TRIP ID** | **Phenotype with *MS1096-GAL4*** | **Interaction with MitoMod** |
| --- | --- | --- | --- | --- | --- | --- |
| *DJ-1α* | Over-expression | *CG6646* | 33603 |  | No | **Enhanced** |
| *DJ-1α* | RNAi | *CG6646* | 38330 | HMS01797 | No | No effect |
| *DJ-1α* | RNAi | *CG6646* | 51177 | HMJ21180 | No | **Enhanced** |
| *DJ-1α* | RNAi | *CG6646* | 39055 | HMS01975 | Yes | Excluded |
|  |  |  |  |  |  |  |
| *DJ-1β* | Over-expression | *CG1349* | 33604 |  | No | **Enhanced** |
| *DJ-1β* | RNAi | *CG1349* | 31261 | JF01202 | No | No effect |
| *DJ-1β* | RNAi | *CG1349* | 38999 | HMS01915 | No | **Enhanced** |
| *DJ-1β* | RNAi | *CG1349* | 38378 | HMS01847 | Yes | Excluded |
|  |  |  |  |  |  |  |
| *Lrrk* | Over-expression | *CG5483* | 35249 |  | No | No effect |
| *Lrrk* | RNAi | *CG5483* | 39019 | HMS01937 | No | **Enhanced** |
| *Lrrk* | RNAi | *CG5483* | 32457 | HMS00456 | No | **Enhanced** |
|  |  |  |  |  |  |  |
| *parkin* | Over-expression | *CG10523* | 51651 |  | No | **Enhanced** |
| *parkin* | RNAi | *CG10523* | 38333 | HMS01800 | No | No effect |
| *parkin* | RNAi | *CG10523* | 31259 | JF01200 | No | No effect |
| *parkin* | RNAi | *CG10523* | 37509 | HMS01651 | Yes | Excluded |
|  |  |  |  |  |  |  |
| *Pink 1* | Over-expression | *CG4523* | 51648 |  | No | **Enhanced** |
| *Pink 1* | RNAi | *CG4523* | 31262 | JF01203 | No | No effect |
| *Pink 1* | RNAi | *CG4523* | 31170 | JF01672 | No | **Enhanced** |
| *Pink 1* | RNAi | *CG4523* | 38262 | HMS01707 | Yes | Excluded |
| *Pink 1* | RNAi | *CG4523* | 41671 | HMS02204 | Yes | Excluded |

*Table S1. Enhancement of MitoMod wing phenotype by knock-down of familial Parkinson’s disease genes*.
